# Temperature variability and common diseases of the elderly in China: a national cross-sectional study

**DOI:** 10.1186/s12940-023-00959-y

**Published:** 2023-01-07

**Authors:** Bo Wen, Bin Bin Su, Jiahui Xue, Junqing Xie, Yao Wu, Li Chen, Yanhui Dong, Xiaolan Wu, Mengfan Wang, Yi Song, Jun Ma, Xiaoying Zheng

**Affiliations:** 1 Institute of Child and Adolescent Health, School of Public Health, Peking University Health Science Center, No 38 Xue Yuan Road, Haidian District, Beijing, 100191 China; 2 Climate, Air Quality Research (CARE) Unit, School of Public Health and Preventive Medicine, Monash University, Level 2, 553 St Kilda Road, Melbourne, VIC 3004 Australia; 3 School of Population Medicine and Public Health, Chinese Academy of Medical Sciences & Peking Union Medical College, No.31, Beijige-3, Dongcheng District, Beijing, 100730 China; 4 First Clinical Medical College of Shanxi Medical University, No. 56 Xinjian South Road, Yingze District, Taiyuan City, 030001 Shanxi Province China; 5 Centre for Statistics in Medicine and NIHR Biomedical Research Centre Oxford, NDORMS, University of Oxford, Oxford, UK; 6 China Research Center on Ageing, 48 Guang ‘anmen South Street, Xicheng District, Beijing, 100054 China; 7 University of Toronto, St.Geogre, 27 King’s College Cir, Toronto, ON M5S Canada

**Keywords:** Temperature variability, Elderly, Diseases, Cardio-cerebrovascular

## Abstract

**Background:**

In the context of climate change, it has been well observed that short-term temperature variability (TV) could increase the overall and cause-specific mortality and morbidity. However, the association between long-term TV and a broader spectrum of diseases is not yet well understood, especially in the elderly.

**Methods:**

Our study used data from the fourth Urban and Rural Elderly Population (UREP) study. Long-term TV was calculated from the standard deviation (SD) of daily minimum and maximum temperatures within the study periods (2010–2014, 2011–2014, 2012–2014, 2013–2014, and 2014). Ten self-reported diseases and conditions were collected by questionnaire, including cataract, hypertension, diabetes, cardio-cerebrovascular diseases, stomach diseases, arthritis, chronic lung disease, asthma, cancer, and reproductive diseases. The province-stratified logistic regression model was used to quantify the association between long-term TV and the prevalence of each disease.

**Results:**

A total of 184,047 participants were included in our study. In general, there were significant associations between TV and the prevalence of most diseases at the national level. Cardio-cerebrovascular disease (OR: 1.16, 95% CI: 1.13, 1.20) generated the highest estimates, followed by stomach diseases (OR: 1.15, 95% CI: 1.10, 1.19), asthma (OR: 1.14, 95% CI: 1.06, 1.22), chronic lung diseases (OR: 1.08, 95% CI: 1.03, 1.13), arthritis (OR: 1.08, 95% CI: 1.05, 1.11), and cataract (OR: 1.06, 95% CI: 1.02, 1.10). Moreover, the associations varied by geographical regions and across subgroups stratified by sex, household income, physical activity, and education.

**Conclusions:**

Our study showed that long-term exposure to TV was associated with the prevalence of main diseases in the elderly. More attention should be paid to the elderly and targeted strategies should be implemented, such as an early warning system.

**Supplementary Information:**

The online version contains supplementary material available at 10.1186/s12940-023-00959-y.

## Background

China has been experiencing unprecedented rapid population aging. In 2018, 17.9% (249.5million) of China’s population were aged over 60 years, which was predicted to be 33.3% by 2050 [1, 2]. Non-communicable diseases (NCDs) have been the leading causes of disability-adjusted life-years (DALYs) in the elderly, such as ischemic heart disease, stroke, and chronic obstructive pulmonary disease (COPD) [3, 4]. In China, the majority of deaths among the elderly could be associated with cardiovascular diseases, neoplasms, and chronic respiratory diseases [5]. The prevalence of NCDs has been associated with multiple factors, such as physical activity, harmful use of tobacco and alcohol, and unhealthy diets [6, 7]. In recent years, increasing attention has been paid to the health impact of environmental factors (e.g., air pollution and non-optimum temperatures) and observed higher mortality/morbidity risks associated with environmental factors among the elderly [8].

Driven by climate change, extreme meteorological events (e.g., heatwave, flood, and thunderstorm) are likely to increase in frequency and severity [9, 10]. It has been well established that long-term or short-term exposure to non-optimum temperatures are associated with the increase of mortality and cardiovascular morbidity [11, 12]. Meanwhile, the short-term temperature variability (TV) was observed to increase the overall and cause-specific mortality and morbidity, and the associations were more evident in the elderly [13–16]. Compared with short-term exposure, the role of long-term TV has been less explored. Although few studies showed an increase in the incidence of respiratory diseases and cardiovascular diseases associated with long-term TV [17–19], the evidence generally confined to cardiopulmonary mortality or morbidity. The association between long-term TV and a broader spectrum of diseases is not yet well understood. Therefore, in this study, we conducted a nationwide cross-sectional study to explore the association between long-term TV and the prevalence of common diseases in about 0.2 million Chinese elderly.

## Methods

### Study population and chronic conditions

**Fig. 1:**
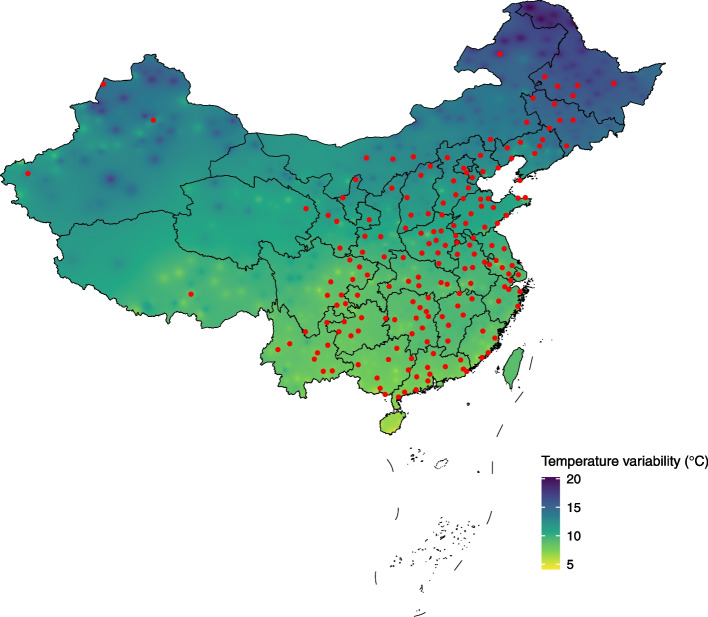
Location of 181 cities included in this study and distribution of TV 2014 in China. TV: temperature variability

Our study used data from the fourth Urban and Rural Elderly Population (UREP) study in 2015 led by the China National Committee on Aging (CNCA), which was a nationally representative survey among the elderly. A multi-stage (provinces, streets or towns, communities or villages) stratified cluster sampling method with probability proportional to size sampling was used to randomly recruit subjects aged above 60 years. In this study, we included a total of 181 cities located in 30 provinces available of meteorological data, which were shown in Fig. 1 and Supplementary Table S1. We ascertained ten self-reported diseases and conditions by asking “Have you been diagnosed with the following conditions: cataract, hypertension, diabetes, cardio-cerebrovascular diseases, stomach diseases, arthritis, chronic lung disease, asthma, cancer, and reproductive diseases”.

### Assessments of covariates

The collection of demographic information was based on self-reported measurements. Education level were categorized into two levels: “illiteracy or primary school” and “high school and above”. Frequency of exercise was categorized into three levels: “never”, “1–5 times per week” and “over 6 times per week”. Marriage status were categorized into two levels: “single” and “married”. We then stratified the participants into three groups according to the tertiles (from the lowest to the highest tertile) of their annual family income. Finally, four climate areas were defined by the quantiles of the city-specific annual mean temperatures from 2010 to 2014 (cold: ≤ 25th; moderate cold: 25th–50th; moderate hot: 50th–75th; hot: >75th) [13].

### Meteorological data

We obtained data on daily minimum temperature, maximum temperature, and relative humidity for each city from the China Meteorological Data Sharing Service Center. Each city has at least one monitoring station. For cities with more than one monitoring station, daily meteorological data were calculated by averaging measurements from all valid monitoring stations in each city. The information of monitoring stations in each city was listed in the Supplementary Table S1.

### Exposure definition

In this study, TV was utilized to represent temperature fluctuations. Long-term TV was calculated as the standard deviation (SD) of daily minimum and maximum temperature (*T*_*min*_ and *T*_*max*_) during the previous 1 to 5 years (2010–2014, 2011–2014, 2012–2014, 2013–2014, and 2014) before the calendar year of the survey (2015) based on previous studies [13, 19–21]. For example, TV 2014 was calculated as SD of *T*_*min*_ and *T*_*max*_ for 365 days in 2014; TV 2010–14 was calculated as SD of *T*_*min*_ and *T*_*max*_ for all days during 2010 and 2014.

### Statistical analysis

We modeled the association of long-term TV with the prevalence of each disease using a province-stratified logistic regression. A binary variable indicating the status of the disease for each participant (present vs. absent) was put into the model as the dependent variable. Province was considered as a stratum variable in our models to account for the potential clustering effect. In the logistic model, a linear function was applied to TV to assess the risk of each disease associated with TV based on previous studies [13, 19, 20, 22]. We also modelled the nonlinear association of TV exposure using a cubic spline with three degrees of freedom, and the relationship tended to be linear when using the nonlinear model (Supplementary material Fig. S5–S9). Different TV exposure (TV 2014, TV 2010–11, TV 2010–12, TV 2010–13, TV 2010–14) were put into model separately. Nonlinear relationships between annual mean temperature as well as relative humidity and prevalence of each disease were controlled using a natural cubic spline with three degrees of freedom (df), as they were identified as potential confounders [22]. Potential demographic and lifestyle confounding factors were also adjusted in the model, including age, sex, urbanity, education level (illiteracy or primary school/high school or higher), exercise (never/1–5 times per week/over 6 times per week), yearly family income (terciles), marriage status (married/single). Finally, the urbanization ratio for each county were included in the model to adjust for the potential impact of socioeconomic status [23]. The association between TV and prevalence of the disease was presented as the odds ratio (OR) with 95% confidence interval (CI) associated with per 1 °C increase in TV exposures.

Analyses were repeated using the data stratified by sex, age groups (60–74 years and above 75 years), urbanity, education level, frequency of exercise, terciles of annual family income, marriage status (married/single) and climatic areas (cold, moderate cold, moderate hot, hot). Random-effect meta-regression fitted by maximum likelihood was used to assess the statistical significance of differences between ORs estimated from each subgroup [24, 25]. To make our results easy to follow, we showed stratified analyses using TV 2014 exposure in the main results and the stratified analyses using other TV exposure periods were shown in supplementary material.

### Sensitivity analysis

Sensitivity analyses were performed to examine the robustness of our results. We changed the df (from 3 to 7) for the nonlinear function of annual mean temperature and relative humidity or replaced the nonlinear function with the linear function. The statistical significance of differences between relative risks estimated from sensitivity analyses and our primary models was assessed using fixed-effect meta-regression model with no statistical adjustment.

We used R software (version 4.0.1) to perform all the analyses, with “mvmeta” package used for the meta-regression.

## Results

**Fig. 2:**
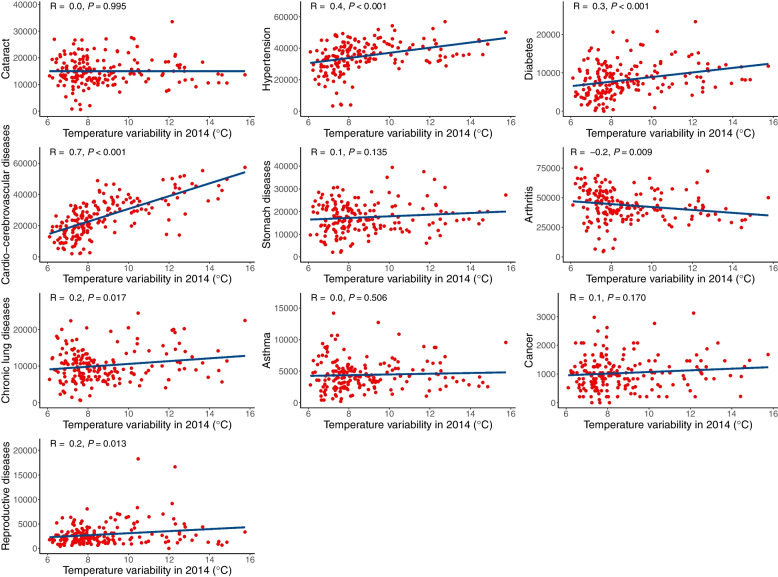
Scatter plot of relationships between TV in 2014 and prevalence of diseases and conditions in China. TV: temperature variability

The distribution of survey sites and their temperature variability is shown in Fig. 1. Generally, distinct region variation of TV was observed in China, with higher TV in the north region compared to that of the south region. Figure 2 shows the scatter plot of the relationship between TV in 2014 and the prevalence of diseases in China. Scatter plots reveal that the prevalence of hypertension, diabetes, cardio-cerebrovascular diseases, chronic lung diseases, and reproductive diseases continuously increased with TV (*p* < 0.05). However, arthritis showed a downward trend with the increase of TV (*p* = 0.009).

The basic characteristics of the study population are shown in Table 1. A total of 184,047 participants were included in our study. The mean age was 69.75 years, and 47.86% were males. Most of the participants are illiterate or with primary school education (70.21%). Nearly half of the participants were living in rural areas (48.00%) and never got any exercise (49.04%). The annual family income was 36.64 [Standard deviation (SD): 40.83] thousand RMB. Approximately 43.47% (80,002) of participants had arthritis, which was the most common disease among the elderly. In descending order of prevalence, the other diseases were hypertension, cardio-cerebrovascular disease, stomach disease, cataract, chronic lung diseases, diabetes, asthma, reproductive diseases, and cancer.

**Table 1:** Basic characteristics of study population

Variables	Characteristics
Overall	184,047
Sex, Male (%)	88,080 (47.86)
Age, years (mean (SD))	69.75 (7.85)
Age group (%)	
60–74	139,672 (75.89)
Over 75	44,375 (24.11)
Rural (%)	88,339 (48.00)
Education (%)	
Illiteracy and primary school	129,222 (70.21)
High school and above	54,249 (29.48)
Missing values	576 (0.31)
Spouse (%)	
Single	50,404 (27.39)
Married	130,901 (71.12)
Missing values	2742 (1.49)
Exercise (%)	
Never	90,258 (49.04)
1–5 times per week	52,563 (28.56)
> 6 times per week	40,252 (21.87)
Missing values	974 (0.53)
Annual family income, RMB (mean (SD))	36,640.44 (40,832.10)
Annual family income, tertiles (RMB, %)	
T1 < 15,000	64,953 (35.29)
T2 15,000–40,000	58,622 (31.85)
T3 > 40,000	57,726 (31.36)
Missing values	2746 (1.49)
Climate areas (%)	
Cold	46,516 (25.27)
Moderate cold	45,622 (24.79)
Moderate hot	47,043 (25.56)
Hot	44,866 (24.38)
Cataract (%)	29,465 (16.01)
Hypertension (%)	68,412 (37.17)
Diabetes (%)	16,297 (8.85)
Cardio-cerebrovascular diseases (%)	49,072 (26.66)
Stomach diseases (%)	32,744 (17.79)
Arthritis (%)	80,002 (43.47)
Chronic lung diseases (%)	18,748 (10.19)
Asthma (%)	8288 (4.50)
Cancer (%)	2127 (1.16)
Reproductive diseases (%)	5165 (2.81)

Table 2 shows the distribution of meteorological factors during the study period. Among 181 cities, the mean TV was 8.62 °C (range: 6.10, 15.75 °C) in 2014. For inter-year variations, TV was similar across different exposure periods, with mean TV ranging between 10.5 °C and 11 °C. Mean temperature and mean relative humidity in 2014 were 15.04 °C (range: 0.55, 23.83 °C) and 69.99% (range: 37.33, 86.54%).

**Table 2:** Distribution of meteorological factors during the study period (2010–2014)

Exposure	Minimum	25th Percentile	Median	Mean	75th Percentile	Maximum
TV 2010–14 (°C)	6.36	9.21	10.34	10.78	11.94	18.29
TV 2011–14 (°C)	6.40	9.29	10.35	10.80	11.94	18.15
TV 2012–14 (°C)	6.27	8.97	10.04	10.58	11.76	17.81
TV 2013–14 (°C)	6.27	9.08	10.19	10.62	11.80	17.52
TV 2014 (°C)	6.10	7.35	7.96	8.62	9.24	15.75
T_mean_ 2010–14 (°C)	−0.36	12.72	15.48	14.68	17.45	23.53
T_mean_ 2011–14 (°C)	−0.29	12.84	15.49	14.70	17.47	23.45
T_mean_ 2012–14 (°C)	−0.34	12.93	15.64	14.79	17.57	23.68
T_mean_ 2013–14 (°C)	0.02	13.19	15.83	15.03	17.82	23.64
T_mean_ 2014 (°C)	0.55	13.41	15.66	15.04	17.45	23.83
RH 2010–14 (%)	38.76	64.30	69.79	68.85	74.90	87.80
RH 2011–14 (%)	39.20	63.84	70.23	68.80	74.76	87.66
RH 2012–14 (%)	39.15	63.79	70.73	69.31	75.08	87.61
RH 2013–14 (%)	39.48	64.23	71.34	69.30	75.56	86.67
RH 2014 (%)	37.33	63.72	73.23	69.99	77.90	86.54

Note: *TV* Temperature variability, *T*_*mean*_ mean temperature, *RH* Mean relative humidity (%)

**Fig. 3:**
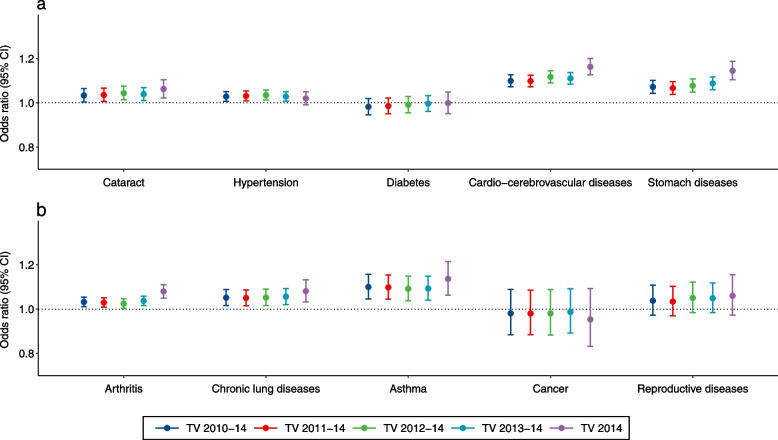
Risk (Odds ratio, OR) for diseases and conditions associated with each 1 °C increase in TV during different exposure periods. TV, temperature variability

Figure 3 shows the odds ratios for the prevalence of all diseases and TV among the elderly. In general, there were significant associations between TV and the prevalence of most diseases at the national level. Examining the associations between TV 2014 and diseases in the elderly, cardio-cerebrovascular disease (OR: 1.16, 95% CI: 1.13, 1.20) generated the highest estimates, followed by stomach diseases (OR: 1.15, 95% CI: 1.10, 1.19), asthma (OR: 1.14, 95% CI: 1.06, 1.22) and chronic lung diseases (OR: 1.08, 95% CI: 1.03, 1.13), arthritis (OR: 1.08, 95% CI: 1.05, 1.11), and cataract (OR: 1.06, 95% CI: 1.02, 1.10). Non-significant associations were observed for hypertension, diabetes, cancer, and reproductive diseases. The results remained robust using different exposure periods.

**Fig. 4:**
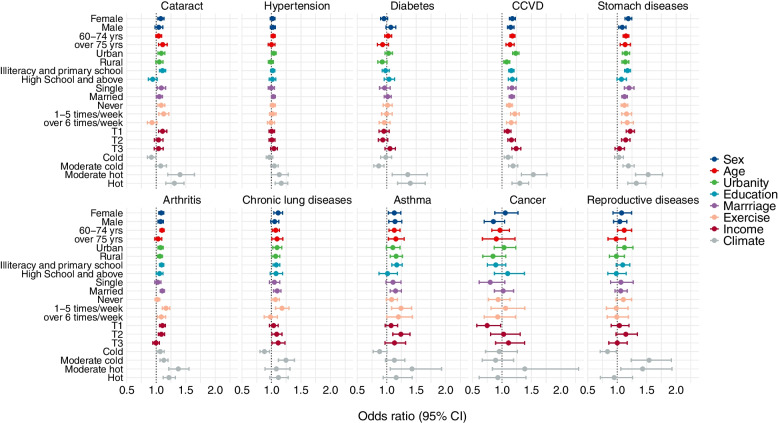
Stratified results of risk (Odds ratio, OR) for diseases and conditions associated with each 1 °C increase in TV during 2014. TV: temperature variability; CCVD: Cardio-cerebrovascular disease

Figure 4 shows the ORs for diseases associated with each 1 °C increase in TV during 2014 in different subgroups. The statistical significance of differences between subgroups was shown in Supplementary Table S2–S6. Compared with males, females showed a significantly higher association between TV and stomach diseases but a significantly lower association between TV and diabetes. Those with higher annual family income were more vulnerable to cardio-cerebrovascular disease and cancer associated with TV. Compared with people who never exercise, individuals who exercised 6 times per week showed a significantly lower association between cataract and TV. A significantly lower association between TV and stomach diseases was observed for those with relatively higher education levels. In addition, relatively higher risks of most diseases associated with TV were shown in hot and moderate hot areas.

The estimations on the associations between long-term TV and each disease remained robust when we changed the df of the nonlinear function of annual mean temperature and relative humidity or replaced the nonlinear function with the linear function (Supplementary Table S7 – S11).

## Discussion

To the best of our knowledge, this is the largest study in China to comprehensively quantify the association between TV and common diseases among the elderly. We found the long-term exposure to TV was significantly associated with the prevalence of most diseases at the national level, including cardio-cerebrovascular disease, stomach diseases, asthma, chronic lung diseases, arthritis, and cataract. The associations varied by geographical regions and across subgroups stratified by sex, household income, physical activity, and education.

In this study, we observed long-term exposure to TV to be most strongly associated with cardio-cerebrovascular diseases, especially for TV 2014. Consistent with our finding, it was reported that the CVD risk increased by 6% with a 1 °C increment of annual TV and the myocardial infarction mortality risk increased by 3.8% with a 1 °C increment in summer TV [18, 20]. A similar relationship can be found in time-series studies, showing that short-term TV exposure could increase the hospitalization and mortality for cardiovascular and cerebrovascular diseases [14, 22, 26–29]. Another common outcome in previous studies associated with TV exposure was chronic lung diseases, and consistent findings were observed by our and previous studies [17, 22]. For example, wintertime temperature variability was significantly associated with the incidence of respiratory diseases in a prospective Chinese elderly cohort in Hongkong [17]. We also observed that mortality risk associated with TV 2014 was relatively higher than that of other exposure periods, which may be related to the potential adaptation abilities of human body to the local climate. For example, it was observed that human body could attain heat acclimation after exposure of heat for several days [30]. Thus, people may be more adapted to the changing temperature within a longer exposure period.

The potential mechanisms behind the relationship between TV and common diseases remain uncertain. One of the hypotheses was that TV may disturb the normal physiological thermoregulation, trigger the autonomic active nervous system and inflammatory reaction, and thus result in adverse impacts on the elderly [18, 31, 32]. Second, the elderly with chronic disease had a higher mortality risk with the increase of TV exposure, which indicated that the elderly may be more susceptible as they experienced a decline in physical function [20, 33]. However, further studies are still warranted to understand the underlying mechanics.

We found greater associations of long-term exposure to TV among females for most of the outcomes, which was supported by previous studies [17, 34, 35]. In comparison, the risk of diabetes with exposure to TV was greater among males, which was inconsistent with the previous study [36]. The differences in sex may be explained by biological differences and different lifestyles, such as diets and physical activity [17]. It could be observed that the risks were relatively lower among those with a higher education level, which could be attributed to the difference in socioeconomic level [37, 38]. Consistent with previous findings, our work suggested that there were greater risks of TV exposure for most of the diseases among those living in hot areas [14]. This finding indicates that people living in warmer areas may be sensitive to temperature variability.

Our study had several strengths. First, this study provided a comprehensive view of the impact of long-term TV exposure on the prevalence of various common diseases among the elderly, which added great value to existing literature. Second, a nationally representative Chinese seniors’ sample and a large number of locations (181 cities) enabled us to provide sufficient power for statistical analyses. Finally, detailed demographic and socioeconomic characteristics were adjusted in this study when examining the association between TV and common diseases.

The study also had some limitations. First, self-reported measure of diseases was used in this study, which could lead to the underestimation of the association between TV and diseases. However, attenuation bias from false-negative reporting could be associated with lower education level and poverty, both of which were adjusted in our analyses and could partly control for the effect of self-reported outcomes on the association between TV and common diseases [39]. Second, we obtained temperature data only from the fixed-site air-monitoring stations in each city and the use of citywide mean TV as proxies for personal exposure is expected to cause exposure misclassification. Third, the level of physical activity was classified by the frequency which may ignore the intensity of exercise and thus lead to potential bias. Besides, a cross-sectional design was used in this study, which cannot provide more detailed information on causal inference. However, given that long-term TV in each city is unlikely to fluctuate wildly and the fact that people have been exposed to TV since birth, this study has the power to assess the association between long-term TV exposure and common diseases.

## Conclusions

In conclusion, our study showed that the long-term exposure to TV was associated with the prevalence of main diseases in the elderly. Our findings suggested that more attention should be paid to the elderly who are more vulnerable to TV in the context of climate change. Further investigation is also needed to help clarify the underlying mechanism behind this relationship.

## Supplementary Information

**Additional file 1: Fig. S1.** Scatter plot of relationship between TV in 2010–14 and prevalence of diseases and conditions in China. TV, temperature variability. **Fig. S2.** Scatter plot of relationship between TV in 2011–14 and prevalence of diseases and conditions in China. TV, temperature variability. 4. **Fig. S3.** Scatter plot of relationship between TV in 2012–14 and prevalence of diseases and conditions in China. TV, temperature variability. **Fig. S4.** Scatter plot of relationship between TV in 2013–14 and prevalence of diseases and conditions in China. TV, temperature variability. **Fig. S5.** Non-linear dose-response relationship between TV in 2010–14 and diseases and conditions in China. TV, temperature variability. **Fig. S6.** Non-linear dose-response relationship between TV in 2011–14 and diseases and conditions in China. TV, temperature variability. **Fig. S7.** Non-linear dose-response relationship between TV in 2012–14 and diseases and conditions in China. TV, temperature variability. **Fig. S8.** Non-linear dose-response relationship between TV in 2013–14 and diseases and conditions in China. TV, temperature variability. **Fig. S9.** Non-linear dose-response relationship between TV in 2014 and diseases and conditions in China. TV, temperature variability. **Table S1**. List of monitoring stations in 181 cities of 30 provinces. **Table S2.** Risk (Odds ratio, OR) for all diseases associated with every 1 °C increase in TV during 2014. **Table S3.** Risk (Odds ratio, OR) for all diseases associated with every 1 °C increase in TV during 2010–2014. **Table S4.** Risk (Odds ratio, OR) for all diseases associated with every 1 °C increase in TV during 2011–2014. **Table S5.** Risk (Odds ratio, OR) for all diseases associated with every 1 °C increase in TV during 2012–2014. **Table S6.** Risk (Odds ratio, OR) for all diseases associated with every 1 °C increase in TV during 2013–2014. **Table S7.** Results of sensitivity analyses for TV 2014 using different df for mean temperature and mean relative humidity. **Table S8.** Results of sensitivity analyses for TV 2010–2014 using different df for mean temperature and mean relative humidity. **Table S9.** Results of sensitivity analyses for TV 2011–2014 using different df for mean temperature and mean relative humidity. **Table S10.** Results of sensitivity analyses for TV 2012–2014 using different df for mean temperature and mean relative humidity. **Table S11.** Results of sensitivity analyses for TV 2013–2014 using different df for mean temperature and mean relative humidity.

## Data Availability

The data that support the findings of this study are available from the China National Committee on Aging (CNCA) but restrictions apply to the availability of these data, which were used under license for the current study, and so are not publicly available. Data are however available from the authors upon reasonable request and with permission of CNCA.
