# Supplementary material for: Temperature variability and common diseases of the elderly in China: a national cross-sectional study
Source: Environ Health. 2023 Jan 7;22:4. doi: 10.1186/s12940-023-00959-y (PMC9824998; doi:10.1186/s12940-023-00959-y)
Supplement: Supplementary file 1 — Additional file 1: Fig. S1. Scatter plot of relationship between TV in 2010–14 and prevalence of diseases and conditions in China. TV, temperature variability. Fig. S2. Scatter plot of relationship between TV in 2011–14 and prevalence of diseases and conditions in China. TV, temperature variability. 4. Fig. S3. Scatter plot of relationship between TV in 2012–14 and prevalence of diseases and conditions in China. TV, temperature variability. Fig. S4. Scatter plot of relationship between TV in 2013–14 and prevalence of diseases and conditions in China. TV, temperature variability. Fig. S5. Non-linear dose-response relationship between TV in 2010–14 and diseases and conditions in China. TV, temperature variability. Fig. S6. Non-linear dose-response relationship between TV in 2011–14 and diseases and conditions in China. TV, temperature variability. Fig. S7. Non-linear dose-response relationship between TV in 2012–14 and diseases and conditions in China. TV, temperature variability. Fig. S8. Non-linear dose-response relationship between TV in 2013–14 and diseases and conditions in China. TV, temperature variability. Fig. S9. Non-linear dose-response relationship between TV in 2014 and diseases and conditions in China. TV, temperature variability. Table S1. List of monitoring stations in 181 cities of 30 provinces. Table S2. Risk (Odds ratio, OR) for all diseases associated with every 1 °C increase in TV during 2014. Table S3. Risk (Odds ratio, OR) for all diseases associated with every 1 °C increase in TV during 2010–2014. Table S4. Risk (Odds ratio, OR) for all diseases associated with every 1 °C increase in TV during 2011–2014. Table S5. Risk (Odds ratio, OR) for all diseases associated with every 1 °C increase in TV during 2012–2014. Table S6. Risk (Odds ratio, OR) for all diseases associated with every 1 °C increase in TV during 2013–2014. Table S7. Results of sensitivity analyses for TV 2014 using different df for mean temperature and mean relative humidity. Table [file 12940_2023_959_MOESM1_ESM.docx]

Temperature variability and common diseases of the elderly in China: a national cross-sectional study

Supplementary material

**Table of Contents**

[Figure S1. Scatter plot of relationship between TV in 2010–14 and prevalence of diseases and conditions in China. TV, temperature variability. 3](#_Toc97908571)

[Figure S2. Scatter plot of relationship between TV in 2011–14 and prevalence of diseases and conditions in China. TV, temperature variability. 4](#_Toc97908572)

[Figure S3. Scatter plot of relationship between TV in 2012–14 and prevalence of diseases and conditions in China. TV, temperature variability. 5](#_Toc97908573)

[Figure S4. Scatter plot of relationship between TV in 2013–14 and prevalence of diseases and conditions in China. TV, temperature variability. 6](#_Toc97908574)

[Figure S5. Non-linear dose-response relationship between TV in 2010–14 and diseases and conditions in China. TV, temperature variability. 7](#_Toc97908575)

[Figure S6. Non-linear dose-response relationship between TV in 2011–14 and diseases and conditions in China. TV, temperature variability. 8](#_Toc97908576)

[Figure S7. Non-linear dose-response relationship between TV in 2012–14 and diseases and conditions in China. TV, temperature variability. 9](#_Toc97908577)

[Figure S8. Non-linear dose-response relationship between TV in 2013–14 and diseases and conditions in China. TV, temperature variability. 10](#_Toc97908578)

[Figure S9. Non-linear dose-response relationship between TV in 2014 and diseases and conditions in China. TV, temperature variability. 11](#_Toc97908579)

[Table S1 List of monitoring stations in 181 cities of 30 provinces. 12](#_Toc97908580)

[Table S2. Risk (Odds ratio, OR) for all diseases associated with every 1℃ increase in TV during 2014. 17](#_Toc97908581)

[Table S3. Risk (Odds ratio, OR) for all diseases associated with every 1℃ increase in TV during 2010–2014. 23](#_Toc97908582)

[Table S4. Risk (Odds ratio, OR) for all diseases associated with every 1℃ increase in TV during 2011–2014. 29](#_Toc97908583)

[Table S5. Risk (Odds ratio, OR) for all diseases associated with every 1℃ increase in TV during 2012–2014. 35](#_Toc97908584)

[Table S6. Risk (Odds ratio, OR) for all diseases associated with every 1℃ increase in TV during 2013–2014. 41](#_Toc97908585)

[Table S7. Results of sensitivity analyses for TV 2014 using different df for mean temperature and mean relative humidity. 47](#_Toc97908586)

[Table S8. Results of sensitivity analyses for TV 2010–2014 using different df for mean temperature and mean relative humidity. 49](#_Toc97908587)

[Table S9. Results of sensitivity analyses for TV 2011–2014 using different df for mean temperature and mean relative humidity. 51](#_Toc97908588)

[Table S10. Results of sensitivity analyses for TV 2012–2014 using different df for mean temperature and mean relative humidity. 53](#_Toc97908589)

[Table S11. Results of sensitivity analyses for TV 2013–2014 using different df for mean temperature and mean relative humidity. 55](#_Toc97908590)


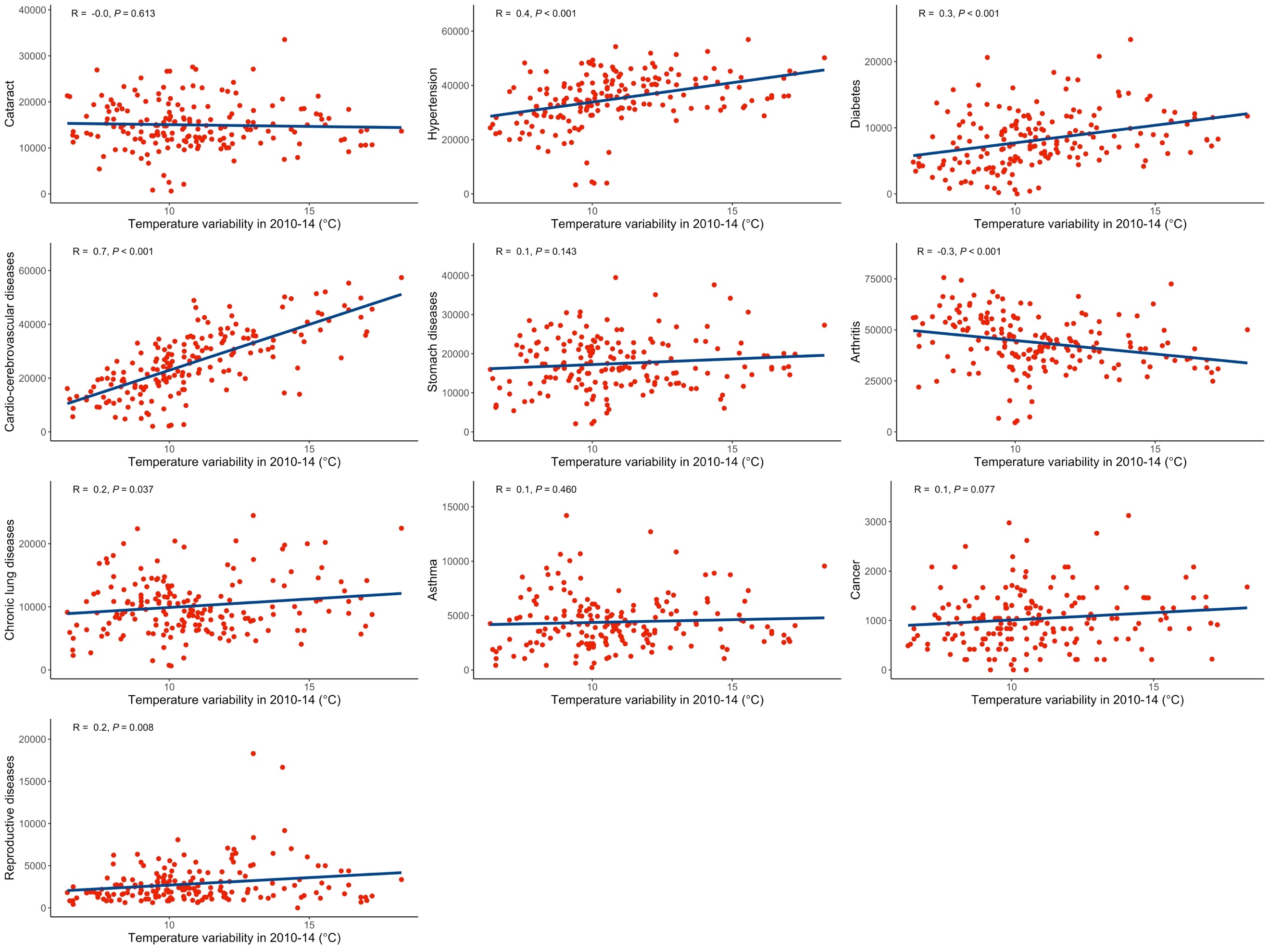


# Figure S1. Scatter plot of relationship between TV in 2010–14 and prevalence of diseases and conditions in China. TV, temperature variability.


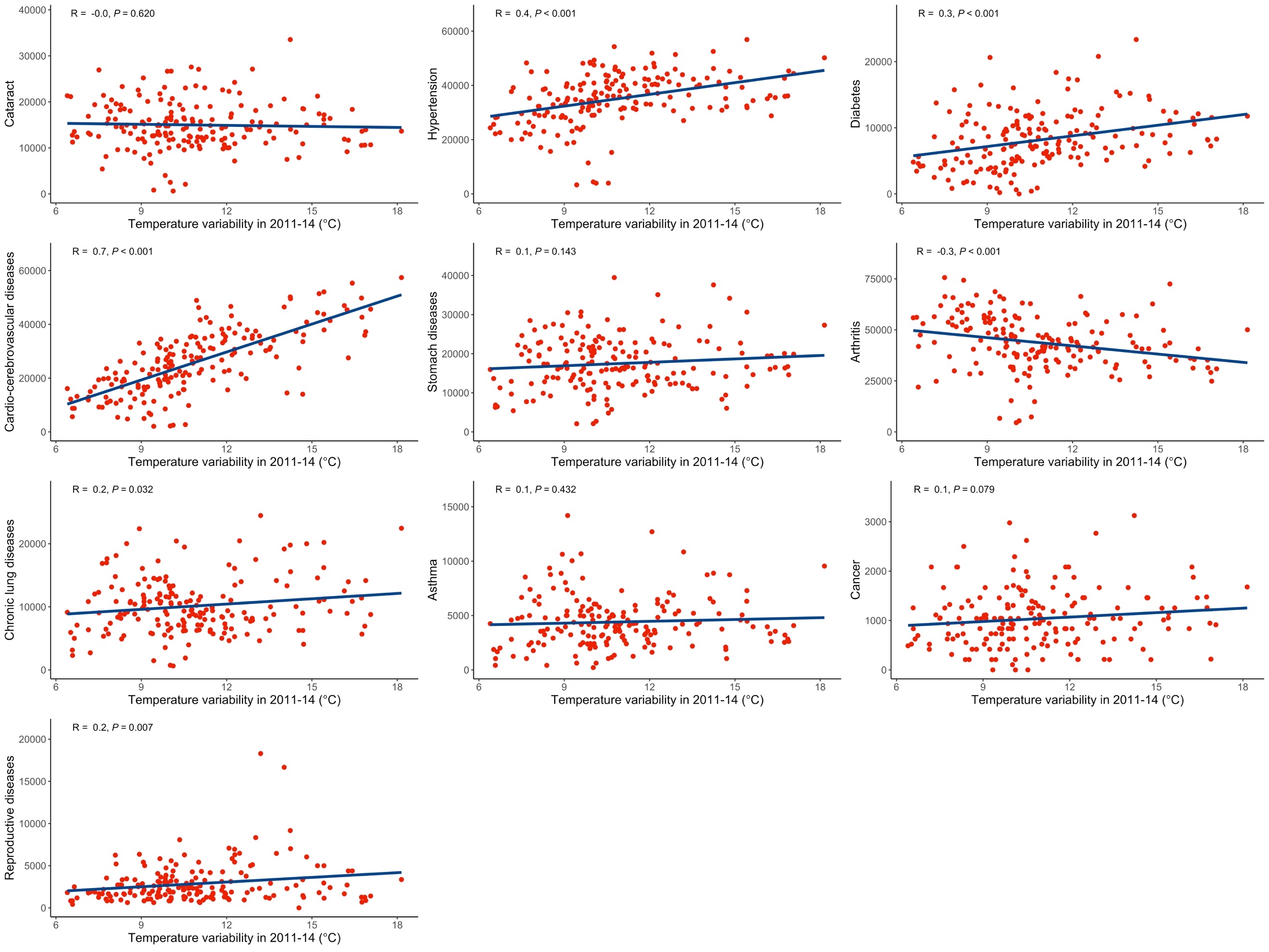


# Figure S2. Scatter plot of relationship between TV in 2011–14 and prevalence of diseases and conditions in China. TV, temperature variability.


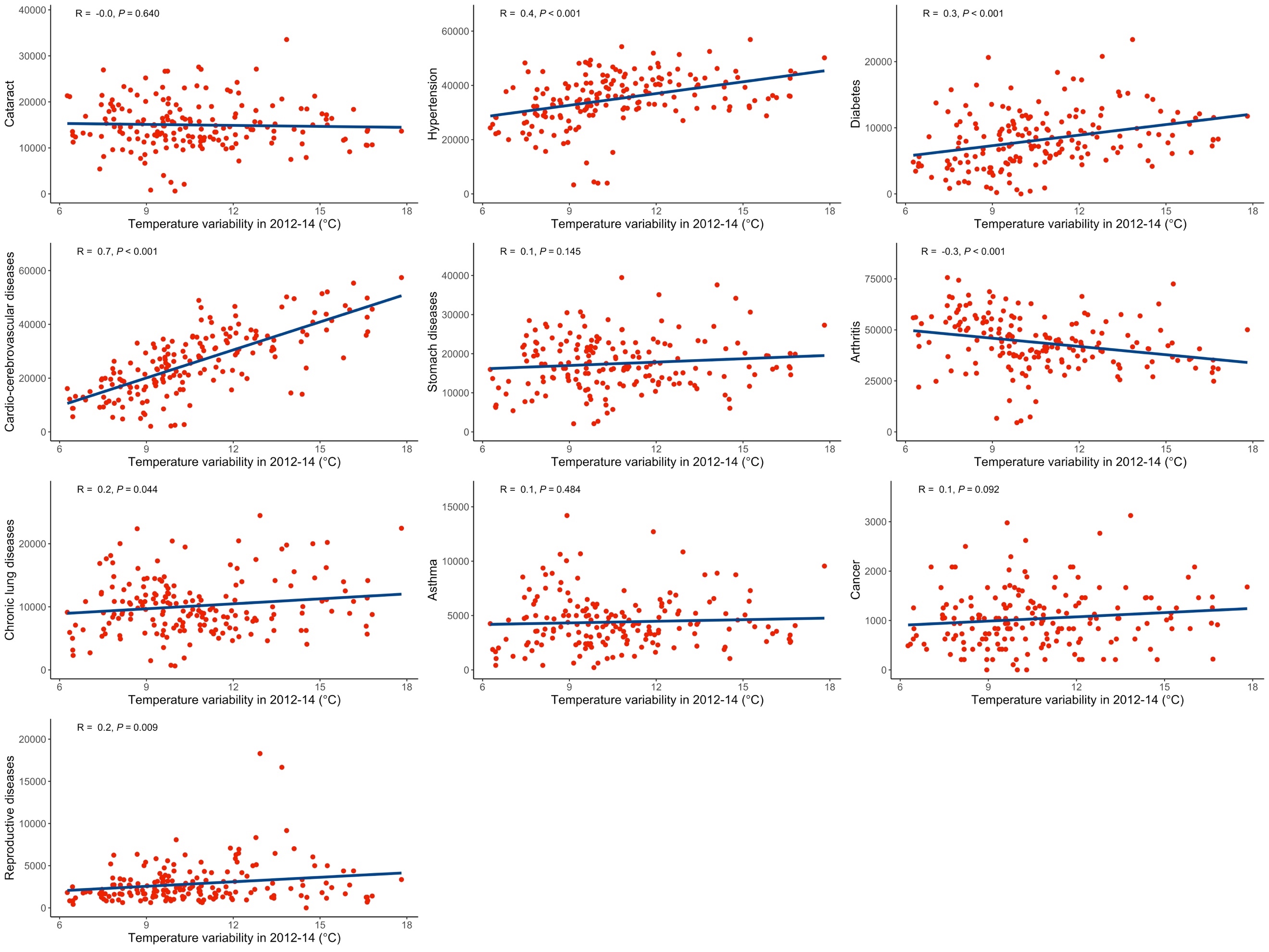


# Figure S3. Scatter plot of relationship between TV in 2012–14 and prevalence of diseases and conditions in China. TV, temperature variability.


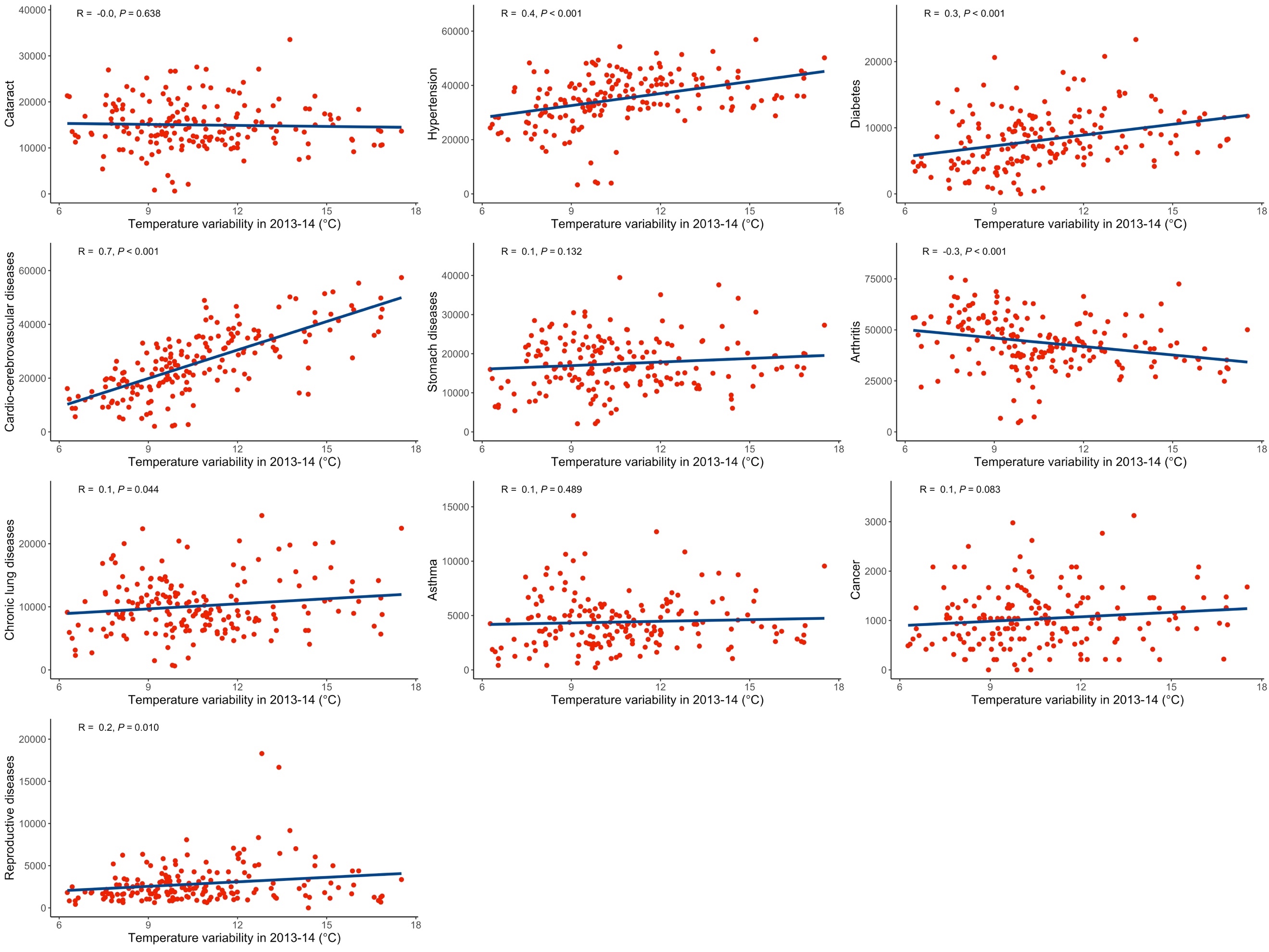


# Figure S4. Scatter plot of relationship between TV in 2013–14 and prevalence of diseases and conditions in China. TV, temperature variability.


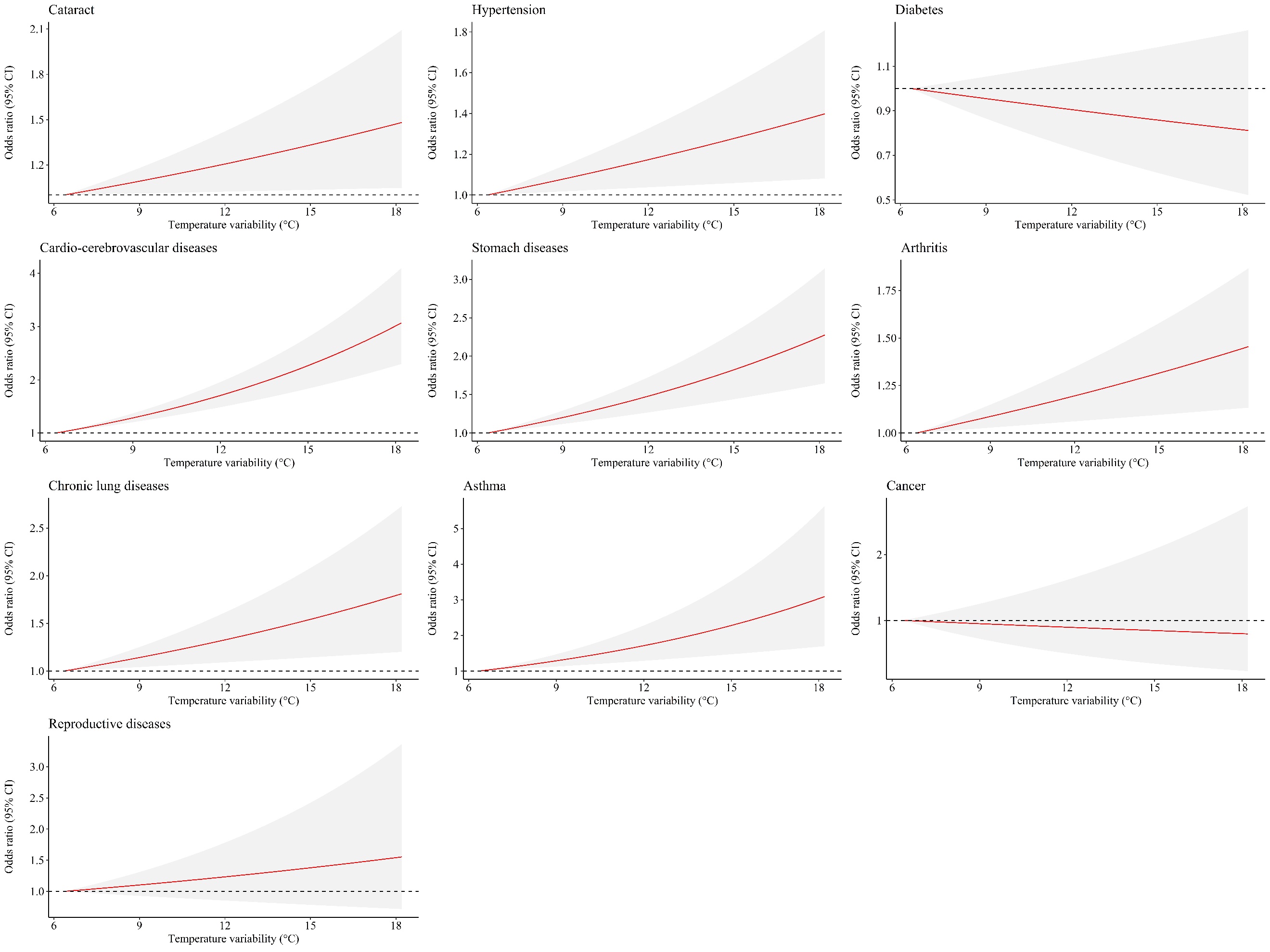


# Figure S5. Non-linear dose-response relationship between TV in 2010–14 and diseases and conditions in China. TV, temperature variability.


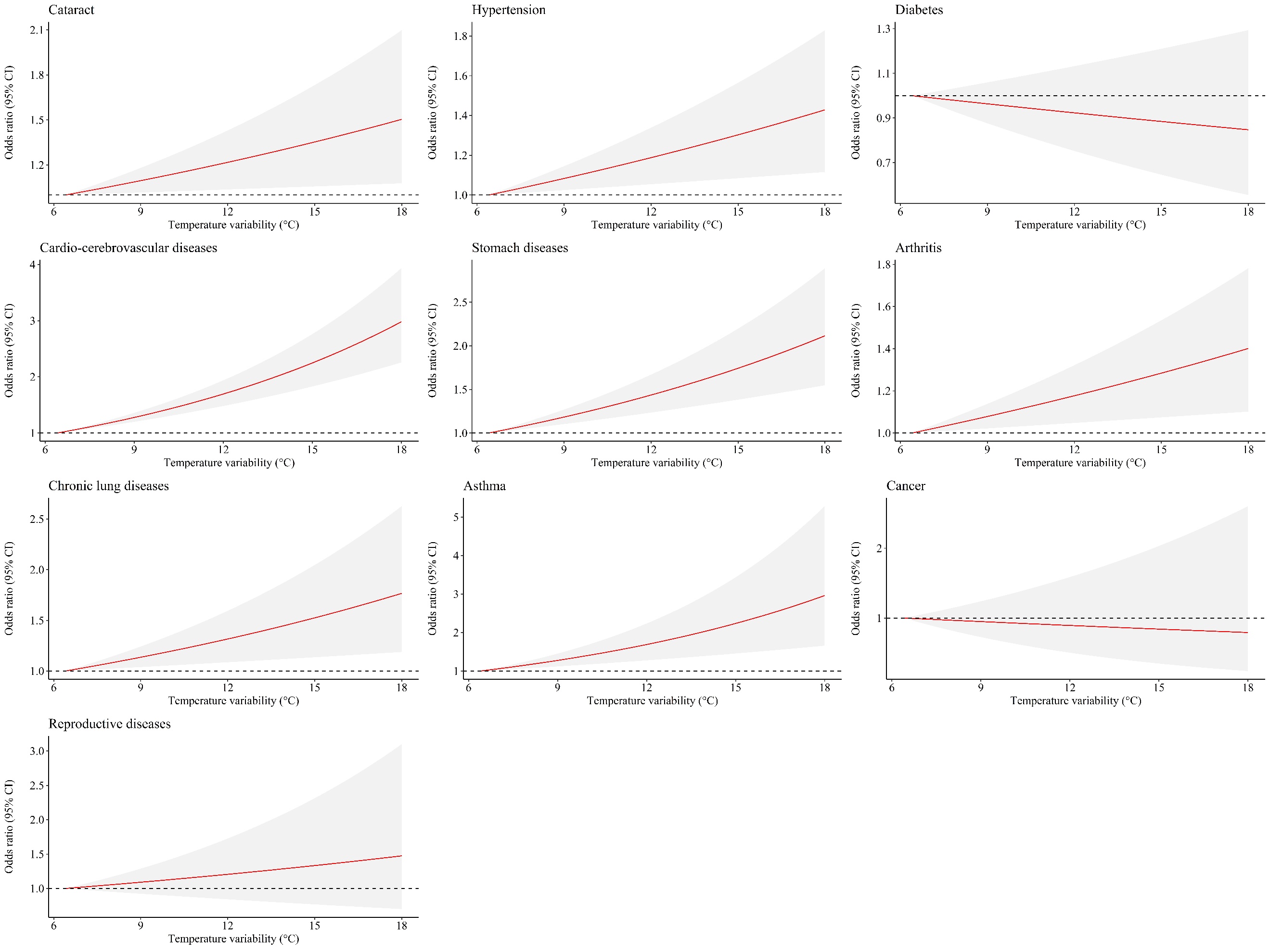


# Figure S6. Non-linear dose-response relationship between TV in 2011–14 and diseases and conditions in China. TV, temperature variability.


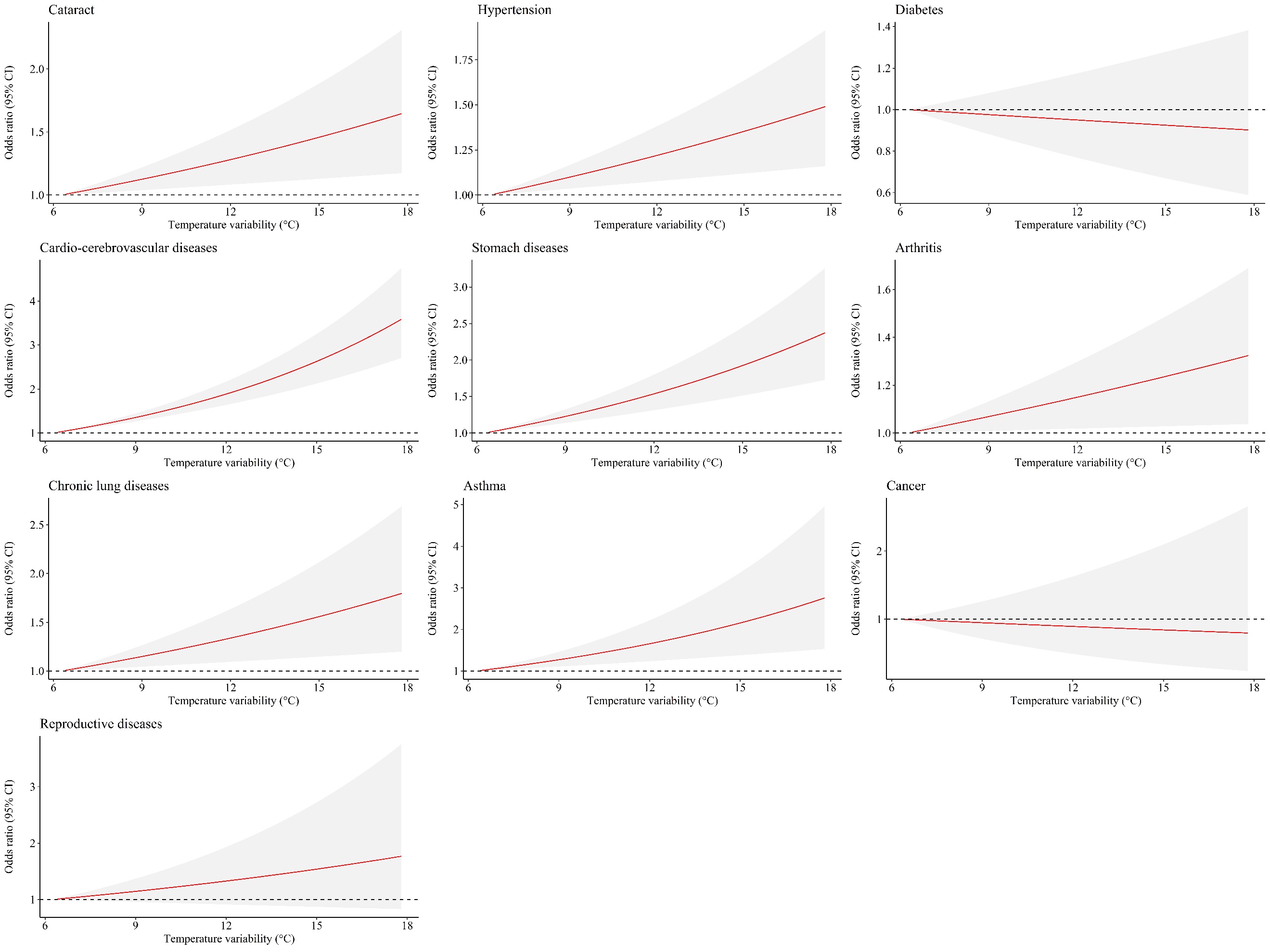


# Figure S7. Non-linear dose-response relationship between TV in 2012–14 and diseases and conditions in China. TV, temperature variability.


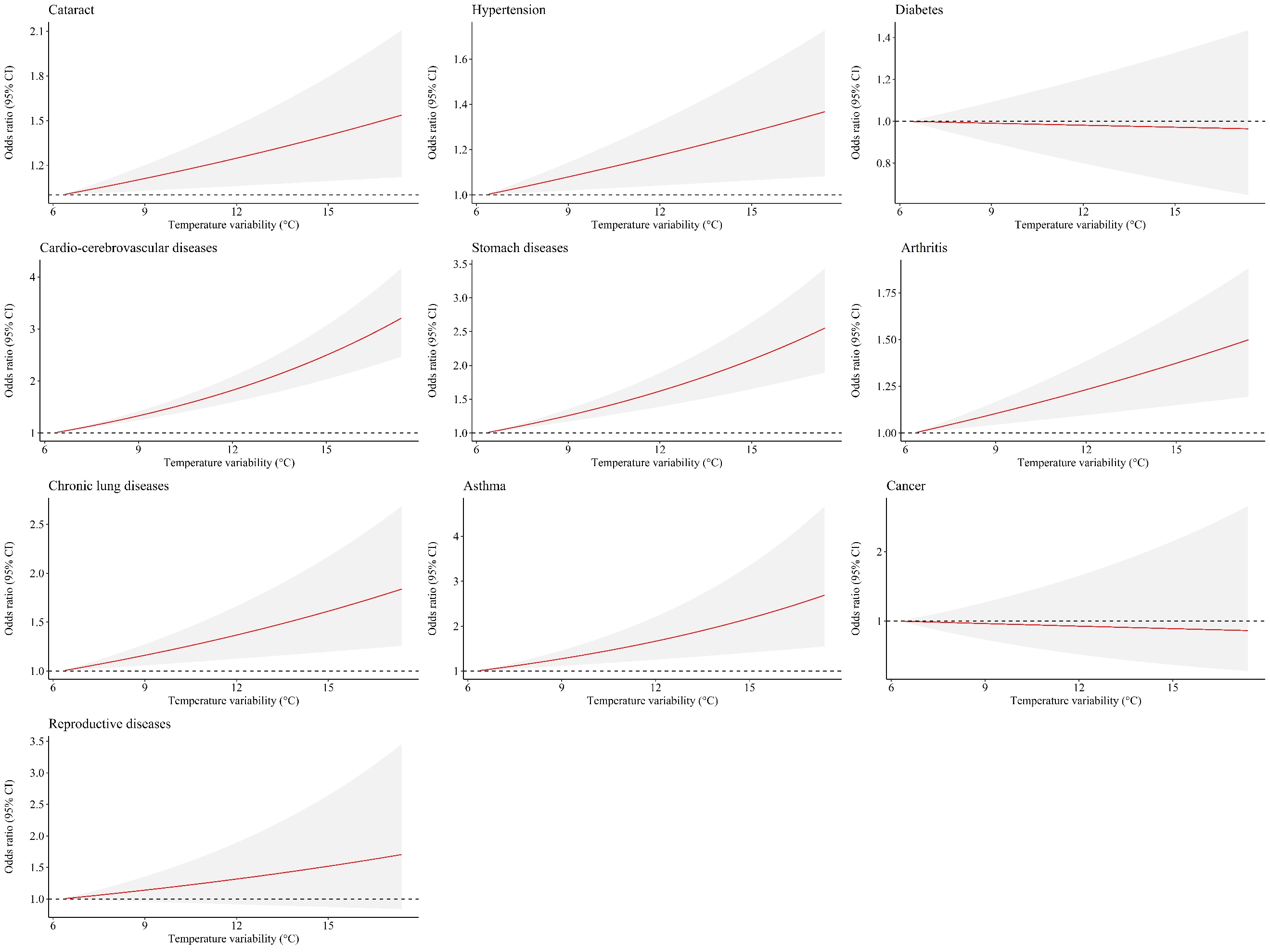


# Figure S8. Non-linear dose-response relationship between TV in 2013–14 and diseases and conditions in China. TV, temperature variability.


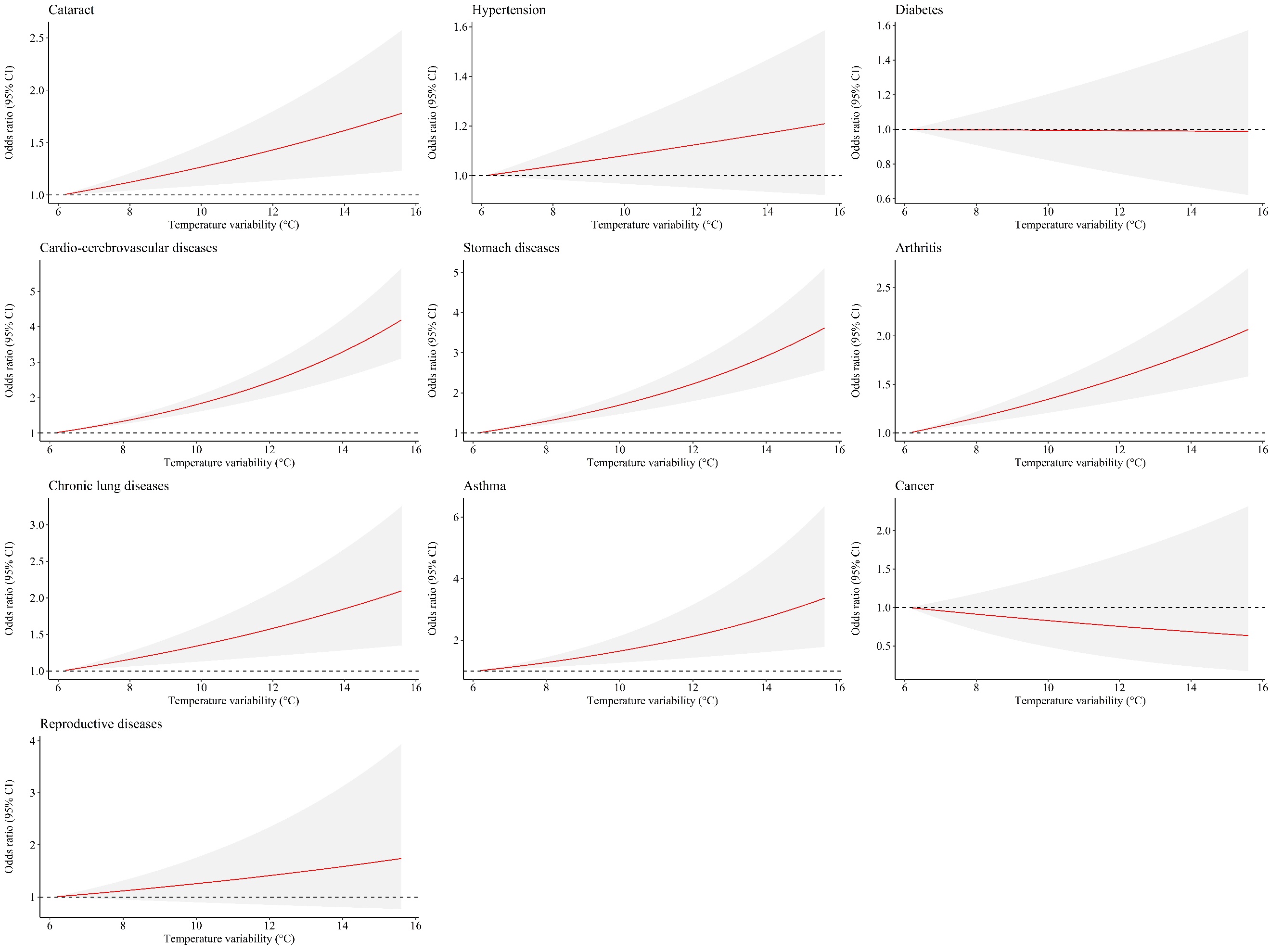


# Figure S9. Non-linear dose-response relationship between TV in 2014 and diseases and conditions in China. TV, temperature variability.

# Table S1 List of monitoring stations in 181 cities of 30 provinces.

| Province | City | Station Number | Station ID |
| --- | --- | --- | --- |
| Anhui | Bozhou | 1 | 58102 |
| Anhui | Liuan | 2 | 58311, 58314 |
| Anhui | Hefei | 2 | 58321, 58326 |
| Anhui | Anqing | 1 | 58319 |
| Anhui | Suzhou | 1 | 58015 |
| Anhui | Chuzhou | 1 | 58236 |
| Anhui | Bengbu | 1 | 58221 |
| Anhui | Fuyang | 1 | 58203 |
| Beijing | Beijing | 2 | 54416, 54511 |
| Chongqing | Chongqing | 5 | 57348, 57432, 57516, 57537, 57633 |
| Fujian | Nanping | 5 | 58725, 58730, 58731, 58734, 58737 |
| Fujian | Xiamen | 1 | 59134 |
| Fujian | Quanzhou | 1 | 58931 |
| Fujian | Fuzhou | 2 | 58847, 58944 |
| Gansu | Lanzhou | 2 | 52884, 52983 |
| Gansu | Dingxi | 3 | 52986, 52996, 56093 |
| Gansu | Qingyang | 1 | 53821 |
| Gansu | Longnan | 1 | 56096 |
| Guangdong | Yunfu | 1 | 59462 |
| Guangdong | Guangzhou | 2 | 59287, 59294 |
| Guangdong | Jieyang | 1 | 59317 |
| Guangdong | Meizhou | 1 | 59117 |
| Guangdong | Shantou | 2 | 59316, 59324 |
| Guangdong | Jiangmen | 2 | 59478, 59673 |
| Guangdong | Heyuan | 2 | 59096, 59293 |
| Guangdong | Qingyuan | 1 | 59072 |
| Guangdong | Zhanjiang | 2 | 59658, 59754 |
| Guangdong | Maoming | 2 | 59456, 59664 |
| Guangdong | Yangjiang | 1 | 59663 |
| Guangdong | Shaoguan | 2 | 57996, 59082 |
| Guangxi | Beihai | 1 | 59644 |
| Guangxi | Nanning | 1 | 59431 |
| Guangxi | Liuzhou | 2 | 57947, 59046 |
| Guangxi | Guilin | 1 | 57957 |
| Guangxi | Wuzhou | 2 | 59058, 59265 |
| Guangxi | Baise | 5 | 59209, 59211, 59218, 59224, 59228 |
| Guangxi | Hezhou | 1 | 59065 |
| Guangxi | Qinzhou | 2 | 59446, 59632 |
| Guizhou | Liupanshui | 1 | 56793 |
| Guizhou | Anshun | 1 | 57806 |
| Guizhou | Bijie | 3 | 56691, 57707, 57803 |
| Guizhou | Guiyang | 1 | 57816 |
| Guizhou | Zunyi | 2 | 57606, 57722 |
| Guizhou | Tongren | 2 | 57731, 57741 |
| Guizhou | Qiandongnan Miao and Dong Autonomous Prefecture | 3 | 57825, 57832, 57932 |
| Guizhou | Qiannan Buyi and Miao Autonomous Prefecture | 2 | 57916, 57922 |
| Hebei | Baoding | 1 | 54602 |
| Hebei | Tangshan | 3 | 54429, 54534, 54539 |
| Hebei | Langfang | 1 | 54518 |
| Hebei | Zhangjiakou | 4 | 53399, 53593, 54401, 54405 |
| Hebei | Chengde | 3 | 54308, 54311, 54423 |
| Hebei | Cangzhou | 3 | 54616, 54618, 54624 |
| Hebei | Qinhuangdao | 2 | 54436, 54449 |
| Hebei | Hengshui | 1 | 54606 |
| Hebei | Xingtai | 2 | 53798, 54705 |
| Heilongjiang | Jiamusi | 2 | 50788, 50873 |
| Heilongjiang | Harbin | 4 | 50877, 50953, 50963, 50968 |
| Heilongjiang | Daqing | 1 | 50950 |
| Heilongjiang | Suihua | 4 | 50756, 50758, 50853, 50854 |
| Heilongjiang | Qiqihar | 4 | 50658, 50742, 50745, 50844 |
| Henan | Xinyang | 2 | 57297, 58208 |
| Henan | Nanyang | 2 | 57156, 57178 |
| Henan | Zhoukou | 1 | 57193 |
| Henan | Shangqiu | 1 | 58005 |
| Henan | Anyang | 1 | 53898 |
| Henan | Pingdingshan | 1 | 57181 |
| Henan | Kaifeng | 1 | 57091 |
| Henan | Luoyang | 1 | 57071 |
| Henan | Xuchang | 1 | 57089 |
| Henan | Zhengzhou | 1 | 57083 |
| Henan | Zhumadian | 1 | 57290 |
| Hubei | Shiyan | 3 | 57251, 57253, 57259 |
| Hubei | Yichang | 2 | 57458, 57461 |
| Hubei | Enshi Tujia and Miao Autonomous Prefecture | 3 | 57355, 57447, 57545 |
| Hubei | Wuhan | 1 | 57494 |
| Hubei | Jingzhou | 1 | 57476 |
| Hubei | Jingmen | 1 | 57378 |
| Hubei | Huanggang | 2 | 57399, 58402 |
| Hunan | Loudi | 1 | 57774 |
| Hunan | Yueyang | 2 | 57584, 57682 |
| Hunan | Changde | 2 | 57562, 57662 |
| Hunan | Huaihua | 3 | 57655, 57745, 57845 |
| Hunan | Zhuzhou | 1 | 57780 |
| Hunan | Yongzhou | 2 | 57866, 57965 |
| Hunan | Xiangxi Tujia and Miao Autonomous Prefecture | 1 | 57649 |
| Hunan | Yiyang | 1 | 57574 |
| Hunan | Hengyang | 3 | 57776, 57872, 57874 |
| Hunan | Chenzhou | 1 | 57972 |
| Hunan | Changsha | 2 | 57679, 57687 |
| Inner Mongolia | Baotou | 3 | 53149, 53352, 53446 |
| Inner Mongolia | Hulun Buir | 8 | 50425, 50434, 50514, 50527, 50548, 50603, 50618, 50639 |
| Inner Mongolia | Hohhot | 1 | 53463 |
| Inner Mongolia | Bayan Nur | 5 | 53083, 53231, 53336, 53420, 53513 |
| Inner Mongolia | Chifeng | 5 | 54027, 54115, 54213, 54218, 54226 |
| Inner Mongolia | Tongliao | 4 | 50834, 54026, 54134, 54135 |
| Jiangsu | Nanjing | 1 | 58238 |
| Jiangsu | Nantong | 1 | 58259 |
| Jiangsu | Changzhou | 1 | 58345 |
| Jiangsu | Xuzhou | 1 | 58027 |
| Jiangsu | Yangzhou | 1 | 58241 |
| Jiangsu | Huaian | 2 | 58138, 58141 |
| Jiangsu | Yancheng | 2 | 58150, 58251 |
| Jiangsu | Suzhou | 1 | 58358 |
| Jiangxi | Jiujiang | 3 | 57598, 58502, 58506 |
| Jiangxi | Nanchang | 1 | 58606 |
| Jiangxi | Jian | 3 | 57799, 57894, 57896 |
| Jiangxi | Yichun | 2 | 57793, 58608 |
| Jiangxi | Fuzhou | 2 | 58715, 58813 |
| Jiangxi | Jingdezhen | 1 | 58527 |
| Jiangxi | Ganzhou | 2 | 57993, 59102 |
| Jilin | Jilin | 5 | 54169, 54171, 54172, 54181, 54273 |
| Jilin | Siping | 2 | 54142, 54157 |
| Jilin | Songyuan | 3 | 50948, 50949, 54049 |
| Jilin | Baicheng | 2 | 50936, 54041 |
| Jilin | Tonghua | 4 | 54263, 54266, 54363, 54377 |
| Jilin | Changchun | 1 | 54161 |
| Liaoning | Dalian | 3 | 54563, 54579, 54584 |
| Liaoning | Shenyang | 2 | 54333, 54342 |
| Liaoning | Huludao | 2 | 54454, 54455 |
| Liaoning | Chaoyang | 1 | 54324 |
| Liaoning | Tieling | 1 | 54254 |
| Liaoning | Fuxin | 1 | 54236 |
| Liaoning | Anshan | 1 | 54339 |
| Ningxia | Zhongwei | 3 | 53704, 53705, 53806 |
| Ningxia | Shizuishan | 2 | 53519, 53615 |
| Qinghai | Xining | 1 | 52866 |
| Shaanxi | Xianyang | 3 | 53929, 57034, 57131 |
| Shaanxi | Ankang | 2 | 57232, 57245 |
| Shaanxi | Baoji | 2 | 57016, 57025 |
| Shaanxi | Yulin | 4 | 53646, 53725, 53740, 53754 |
| Shaanxi | Hanzhong | 3 | 57106, 57127, 57134 |
| Shandong | Dongying | 2 | 54736, 54744 |
| Shandong | Linyi | 2 | 54929, 54938 |
| Shandong | Weihai | 1 | 54776 |
| Shandong | Dezhou | 2 | 54714, 54715 |
| Shandong | Rizhao | 2 | 54936, 54945 |
| Shandong | Taian | 2 | 54826, 54827 |
| Shandong | Jining | 1 | 54916 |
| Shandong | Zibo | 3 | 54824, 54830, 54836 |
| Shandong | Binzhou | 1 | 54725 |
| Shandong | Weifang | 1 | 54843 |
| Shandong | Yantai | 6 | 54751, 54753, 54764, 54765, 54852, 54863 |
| Shandong | Heze | 2 | 54906, 54909 |
| Shandong | Qingdao | 1 | 54857 |
| Shanghai | Shanghai | 2 | 58362, 58367 |
| Shanxi | Linfen | 3 | 53853, 53868, 53963 |
| Shanxi | Lvliang | 2 | 53664, 53764 |
| Shanxi | Datong | 1 | 53487 |
| Shanxi | Shuozhou | 1 | 53478 |
| Shanxi | Yuncheng | 1 | 53959 |
| Shanxi | Changzhi | 1 | 53882 |
| Sichuan | Leshan | 2 | 56385, 56386 |
| Sichuan | Neijing | 1 | 57503 |
| Sichuan | Nanchong | 2 | 57306, 57411 |
| Sichuan | Yibin | 1 | 56492 |
| Sichuan | Bazhong | 1 | 57313 |
| Sichuan | Guangyuan | 1 | 57206 |
| Sichuan | Chengdu | 2 | 56187, 56188 |
| Sichuan | Panzhihua | 2 | 56666, 56674 |
| Sichuan | Luzhou | 1 | 57608 |
| Sichuan | Dazhou | 1 | 57237 |
| Tianjin | Tianjin | 2 | 54527, 54623 |
| Tibet | Lhasa | 1 | 55493 |
| Xinjiang | Urumqi | 1 | 51477 |
| Xinjiang | Tulufan | 2 | 51526, 51573 |
| Xinjiang | Kashgar Prefecture | 2 | 51709, 51811 |
| Xinjiang | Tarbagatay Prefecture | 3 | 51133, 51156, 51241 |
| Yunnan | Baoshan | 2 | 56739, 56748 |
| Yunnan | Dali Bai Autonomous Prefecture | 1 | 56751 |
| Yunnan | Wenshan Zhuang and Miao Autonomous Prefecture | 2 | 56991, 59007 |
| Yunnan | Kunming | 2 | 56778, 56880 |
| Yunnan | Zhaotong | 1 | 56586 |
| Yunnan | Qujing | 1 | 56684 |
| Yunnan | Yuxi | 2 | 56875, 56966 |
| Yunnan | Hani-Yi Autonomous Prefecture of Honghe | 1 | 56886 |
| Zhejiang | Taizhou | 4 | 58660, 58665, 58666, 58667 |
| Zhejiang | Jiaxing | 1 | 58464 |
| Zhejiang | Ningbo | 3 | 58467, 58562, 58569 |
| Zhejiang | Hangzhou | 2 | 58457, 58543 |
| Zhejiang | Shaoxing | 1 | 58556 |
| Zhejiang | Jinhua | 1 | 58549 |

# Table S2. Risk (Odds ratio, OR) for all diseases associated with every 1℃ increase in TV during 2014.

| Disease | Strata | Groups | Odds ratio (95% CI) | P for difference |
| --- | --- | --- | --- | --- |
| Cataract | Sex | Female | 1.08 (1.03, 1.13) | Ref |
| Cataract | Sex | Male | 1.04 (0.98, 1.11) | 0.418 |
| Cataract | Age | 60–74 yrs | 1.04 (0.99, 1.09) | Ref |
| Cataract | Age | over 75 yrs | 1.11 (1.04, 1.18) | 0.125 |
| Cataract | Urbanity | Urban | 1.09 (1.03, 1.15) | Ref |
| Cataract | Urbanity | Rural | 1.05 (0.99, 1.12) | 0.436 |
| Cataract | Education | Illiteracy and primary school | 1.11 (1.06, 1.16) | Ref |
| Cataract | Education | High School and above | 0.94 (0.87, 1.02) | <0.001 |
| Cataract | Marriage | Single | 1.09 (1.02, 1.16) | Ref |
| Cataract | Marriage | Married | 1.05 (1.00, 1.10) | 0.444 |
| Cataract | Exercise | Never | 1.08 (1.03, 1.14) | Ref |
| Cataract | Exercise | 1–5 times/week | 1.13 (1.04, 1.21) | 0.436 |
| Cataract | Exercise | over 6 times/week | 0.93 (0.85, 1.01) | 0.003 |
| Cataract | Income | T1 | 1.11 (1.04, 1.18) | Ref |
| Cataract | Income | T2 | 1.04 (0.97, 1.11) | 0.184 |
| Cataract | Income | T3 | 1.04 (0.96, 1.12) | 0.203 |
| Cataract | Climate | Cold | 0.92 (0.85, 0.99) | Ref |
| Cataract | Climate | Moderate cold | 1.08 (0.99, 1.17) | 0.007 |
| Cataract | Climate | Moderate hot | 1.40 (1.20, 1.65) | <0.001 |
| Cataract | Climate | Hot | 1.31 (1.16, 1.47) | <0.001 |
| Hypertension | Sex | Female | 1.02 (0.98, 1.06) | Ref |
| Hypertension | Sex | Male | 1.02 (0.98, 1.07) | 0.866 |
| Hypertension | Age | 60–74 yrs | 1.03 (0.99, 1.06) | Ref |
| Hypertension | Age | over 75 yrs | 1.00 (0.94, 1.06) | 0.407 |
| Hypertension | Urbanity | Urban | 1.04 (1.00, 1.08) | Ref |
| Hypertension | Urbanity | Rural | 0.99 (0.95, 1.03) | 0.107 |
| Hypertension | Education | Illiteracy and primary school | 1.02 (0.99, 1.06) | Ref |
| Hypertension | Education | High School and above | 1.02 (0.96, 1.07) | 0.907 |
| Hypertension | Marriage | Single | 1.00 (0.94, 1.05) | Ref |
| Hypertension | Marriage | Married | 1.03 (1.00, 1.07) | 0.263 |
| Hypertension | Exercise | Never | 1.02 (0.98, 1.07) | Ref |
| Hypertension | Exercise | 1–5 times/week | 1.02 (0.97, 1.08) | 0.941 |
| Hypertension | Exercise | over 6 times/week | 0.99 (0.93, 1.05) | 0.403 |
| Hypertension | Income | T1 | 1.00 (0.96, 1.05) | Ref |
| Hypertension | Income | T2 | 1.01 (0.96, 1.06) | 0.934 |
| Hypertension | Income | T3 | 1.04 (0.99, 1.10) | 0.328 |
| Hypertension | Climate | Cold | 0.96 (0.91, 1.02) | Ref |
| Hypertension | Climate | Moderate cold | 1.05 (0.99, 1.11) | 0.05 |
| Hypertension | Climate | Moderate hot | 1.13 (1.00, 1.28) | 0.022 |
| Hypertension | Climate | Hot | 1.17 (1.06, 1.28) | 0.001 |
| Diabetes | Sex | Female | 0.95 (0.90, 1.02) | Ref |
| Diabetes | Sex | Male | 1.07 (0.99, 1.16) | 0.025 |
| Diabetes | Age | 60–74 yrs | 1.02 (0.97, 1.08) | Ref |
| Diabetes | Age | over 75 yrs | 0.93 (0.84, 1.03) | 0.104 |
| Diabetes | Urbanity | Urban | 1.03 (0.97, 1.10) | Ref |
| Diabetes | Urbanity | Rural | 0.92 (0.85, 1.01) | 0.04 |
| Diabetes | Education | Illiteracy and primary school | 0.98 (0.92, 1.04) | Ref |
| Diabetes | Education | High School and above | 1.04 (0.96, 1.13) | 0.259 |
| Diabetes | Marriage | Single | 0.96 (0.88, 1.06) | Ref |
| Diabetes | Marriage | Married | 1.01 (0.96, 1.07) | 0.363 |
| Diabetes | Exercise | Never | 1.01 (0.94, 1.09) | Ref |
| Diabetes | Exercise | 1–5 times/week | 1.00 (0.91, 1.09) | 0.821 |
| Diabetes | Exercise | over 6 times/week | 0.96 (0.87, 1.06) | 0.379 |
| Diabetes | Income | T1 | 0.95 (0.87, 1.04) | Ref |
| Diabetes | Income | T2 | 0.93 (0.86, 1.02) | 0.764 |
| Diabetes | Income | T3 | 1.06 (0.97, 1.15) | 0.1 |
| Diabetes | Climate | Cold | 0.98 (0.90, 1.08) | Ref |
| Diabetes | Climate | Moderate cold | 0.86 (0.78, 0.95) | 0.061 |
| Diabetes | Climate | Moderate hot | 1.36 (1.09, 1.69) | 0.008 |
| Diabetes | Climate | Hot | 1.40 (1.19, 1.65) | <0.001 |
| Cardio-cerebrovascular diseases | Sex | Female | 1.18 (1.13, 1.23) | Ref |
| Cardio-cerebrovascular diseases | Sex | Male | 1.15 (1.09, 1.20) | 0.461 |
| Cardio-cerebrovascular diseases | Age | 60–74 yrs | 1.18 (1.14, 1.22) | Ref |
| Cardio-cerebrovascular diseases | Age | over 75 yrs | 1.14 (1.07, 1.21) | 0.322 |
| Cardio-cerebrovascular diseases | Urbanity | Urban | 1.23 (1.18, 1.29) | Ref |
| Cardio-cerebrovascular diseases | Urbanity | Rural | 1.08 (1.03, 1.13) | <0.001 |
| Cardio-cerebrovascular diseases | Education | Illiteracy and primary school | 1.16 (1.12, 1.20) | Ref |
| Cardio-cerebrovascular diseases | Education | High School and above | 1.18 (1.11, 1.25) | 0.679 |
| Cardio-cerebrovascular diseases | Marriage | Single | 1.17 (1.10, 1.24) | Ref |
| Cardio-cerebrovascular diseases | Marriage | Married | 1.17 (1.12, 1.21) | 0.945 |
| Cardio-cerebrovascular diseases | Exercise | Never | 1.13 (1.08, 1.18) | Ref |
| Cardio-cerebrovascular diseases | Exercise | 1–5 times/week | 1.22 (1.15, 1.29) | 0.044 |
| Cardio-cerebrovascular diseases | Exercise | over 6 times/week | 1.16 (1.08, 1.24) | 0.541 |
| Cardio-cerebrovascular diseases | Income | T1 | 1.10 (1.04, 1.15) | Ref |
| Cardio-cerebrovascular diseases | Income | T2 | 1.16 (1.10, 1.23) | 0.143 |
| Cardio-cerebrovascular diseases | Income | T3 | 1.24 (1.17, 1.32) | 0.003 |
| Cardio-cerebrovascular diseases | Climate | Cold | 1.10 (1.04, 1.17) | Ref |
| Cardio-cerebrovascular diseases | Climate | Moderate cold | 1.19 (1.11, 1.27) | 0.104 |
| Cardio-cerebrovascular diseases | Climate | Moderate hot | 1.53 (1.33, 1.77) | <0.001 |
| Cardio-cerebrovascular diseases | Climate | Hot | 1.30 (1.17, 1.45) | 0.009 |
| Stomach diseases | Sex | Female | 1.19 (1.13, 1.25) | Ref |
| Stomach diseases | Sex | Male | 1.09 (1.03, 1.15) | 0.016 |
| Stomach diseases | Age | 60–74 yrs | 1.15 (1.10, 1.20) | Ref |
| Stomach diseases | Age | over 75 yrs | 1.13 (1.05, 1.22) | 0.771 |
| Stomach diseases | Urbanity | Urban | 1.15 (1.09, 1.21) | Ref |
| Stomach diseases | Urbanity | Rural | 1.14 (1.08, 1.20) | 0.784 |
| Stomach diseases | Education | Illiteracy and primary school | 1.17 (1.13, 1.23) | Ref |
| Stomach diseases | Education | High School and above | 1.07 (0.99, 1.15) | 0.036 |
| Stomach diseases | Marriage | Single | 1.20 (1.12, 1.29) | Ref |
| Stomach diseases | Marriage | Married | 1.12 (1.08, 1.17) | 0.109 |
| Stomach diseases | Exercise | Never | 1.12 (1.07, 1.18) | Ref |
| Stomach diseases | Exercise | 1–5 times/week | 1.16 (1.08, 1.24) | 0.474 |
| Stomach diseases | Exercise | over 6 times/week | 1.17 (1.07, 1.28) | 0.414 |
| Stomach diseases | Income | T1 | 1.22 (1.15, 1.29) | Ref |
| Stomach diseases | Income | T2 | 1.14 (1.07, 1.22) | 0.136 |
| Stomach diseases | Income | T3 | 1.04 (0.96, 1.12) | 0.001 |
| Stomach diseases | Climate | Cold | 1.03 (0.96, 1.11) | Ref |
| Stomach diseases | Climate | Moderate cold | 1.19 (1.10, 1.29) | 0.008 |
| Stomach diseases | Climate | Moderate hot | 1.53 (1.31, 1.77) | <0.001 |
| Stomach diseases | Climate | Hot | 1.32 (1.18, 1.48) | <0.001 |
| Arthritis | Sex | Female | 1.09 (1.04, 1.13) | Ref |
| Arthritis | Sex | Male | 1.08 (1.03, 1.12) | 0.743 |
| Arthritis | Age | 60–74 yrs | 1.10 (1.06, 1.13) | Ref |
| Arthritis | Age | over 75 yrs | 1.03 (0.98, 1.09) | 0.068 |
| Arthritis | Urbanity | Urban | 1.07 (1.03, 1.12) | Ref |
| Arthritis | Urbanity | Rural | 1.06 (1.02, 1.11) | 0.721 |
| Arthritis | Education | Illiteracy and primary school | 1.09 (1.06, 1.13) | Ref |
| Arthritis | Education | High School and above | 1.06 (1.00, 1.12) | 0.312 |
| Arthritis | Marriage | Single | 1.03 (0.97, 1.08) | Ref |
| Arthritis | Marriage | Married | 1.10 (1.07, 1.14) | 0.026 |
| Arthritis | Exercise | Never | 1.02 (0.98, 1.06) | Ref |
| Arthritis | Exercise | 1–5 times/week | 1.17 (1.11, 1.23) | <0.001 |
| Arthritis | Exercise | over 6 times/week | 1.09 (1.02, 1.16) | 0.094 |
| Arthritis | Income | T1 | 1.11 (1.06, 1.16) | Ref |
| Arthritis | Income | T2 | 1.09 (1.04, 1.14) | 0.631 |
| Arthritis | Income | T3 | 1.00 (0.94, 1.05) | 0.005 |
| Arthritis | Climate | Cold | 1.07 (1.01, 1.13) | Ref |
| Arthritis | Climate | Moderate cold | 1.13 (1.06, 1.20) | 0.203 |
| Arthritis | Climate | Moderate hot | 1.37 (1.21, 1.56) | <0.001 |
| Arthritis | Climate | Hot | 1.22 (1.12, 1.32) | 0.014 |
| Chronic lung diseases | Sex | Female | 1.11 (1.04, 1.19) | Ref |
| Chronic lung diseases | Sex | Male | 1.05 (0.99, 1.12) | 0.251 |
| Chronic lung diseases | Age | 60–74 yrs | 1.07 (1.01, 1.13) | Ref |
| Chronic lung diseases | Age | over 75 yrs | 1.09 (1.00, 1.19) | 0.695 |
| Chronic lung diseases | Urbanity | Urban | 1.10 (1.02, 1.17) | Ref |
| Chronic lung diseases | Urbanity | Rural | 1.07 (1.00, 1.14) | 0.633 |
| Chronic lung diseases | Education | Illiteracy and primary school | 1.08 (1.03, 1.14) | Ref |
| Chronic lung diseases | Education | High School and above | 1.08 (0.98, 1.19) | 0.942 |
| Chronic lung diseases | Marriage | Single | 1.05 (0.97, 1.14) | Ref |
| Chronic lung diseases | Marriage | Married | 1.10 (1.04, 1.16) | 0.382 |
| Chronic lung diseases | Exercise | Never | 1.07 (1.01, 1.14) | Ref |
| Chronic lung diseases | Exercise | 1–5 times/week | 1.18 (1.07, 1.30) | 0.09 |
| Chronic lung diseases | Exercise | over 6 times/week | 0.98 (0.88, 1.10) | 0.205 |
| Chronic lung diseases | Income | T1 | 1.04 (0.97, 1.11) | Ref |
| Chronic lung diseases | Income | T2 | 1.09 (1.00, 1.18) | 0.379 |
| Chronic lung diseases | Income | T3 | 1.11 (1.01, 1.23) | 0.254 |
| Chronic lung diseases | Climate | Cold | 0.88 (0.80, 0.96) | Ref |
| Chronic lung diseases | Climate | Moderate cold | 1.25 (1.12, 1.39) | <0.001 |
| Chronic lung diseases | Climate | Moderate hot | 1.08 (0.89, 1.32) | 0.061 |
| Chronic lung diseases | Climate | Hot | 1.12 (0.97, 1.28) | 0.005 |
| Asthma | Sex | Female | 1.13 (1.03, 1.24) | Ref |
| Asthma | Sex | Male | 1.14 (1.04, 1.26) | 0.874 |
| Asthma | Age | 60–74 yrs | 1.13 (1.04, 1.23) | Ref |
| Asthma | Age | over 75 yrs | 1.15 (1.03, 1.30) | 0.749 |
| Asthma | Urbanity | Urban | 1.10 (0.99, 1.22) | Ref |
| Asthma | Urbanity | Rural | 1.16 (1.06, 1.27) | 0.482 |
| Asthma | Education | Illiteracy and primary school | 1.17 (1.09, 1.26) | Ref |
| Asthma | Education | High School and above | 1.01 (0.87, 1.18) | 0.099 |
| Asthma | Marriage | Single | 1.11 (0.99, 1.24) | Ref |
| Asthma | Marriage | Married | 1.15 (1.06, 1.25) | 0.599 |
| Asthma | Exercise | Never | 1.08 (0.99, 1.18) | Ref |
| Asthma | Exercise | 1–5 times/week | 1.24 (1.08, 1.42) | 0.095 |
| Asthma | Exercise | over 6 times/week | 1.20 (1.00, 1.44) | 0.32 |
| Asthma | Income | T1 | 1.07 (0.97, 1.19) | Ref |
| Asthma | Income | T2 | 1.24 (1.10, 1.40) | 0.068 |
| Asthma | Income | T3 | 1.13 (0.97, 1.32) | 0.573 |
| Asthma | Climate | Cold | 0.88 (0.77, 1.01) | Ref |
| Asthma | Climate | Moderate cold | 1.13 (0.98, 1.31) | 0.013 |
| Asthma | Climate | Moderate hot | 1.43 (1.06, 1.93) | 0.004 |
| Asthma | Climate | Hot | 1.16 (0.94, 1.43) | 0.029 |
| Cancer | Sex | Female | 1.06 (0.88, 1.27) | Ref |
| Cancer | Sex | Male | 0.85 (0.70, 1.05) | 0.129 |
| Cancer | Age | 60–74 yrs | 0.97 (0.83, 1.13) | Ref |
| Cancer | Age | over 75 yrs | 0.90 (0.67, 1.22) | 0.703 |
| Cancer | Urbanity | Urban | 1.04 (0.87, 1.23) | Ref |
| Cancer | Urbanity | Rural | 0.85 (0.68, 1.06) | 0.173 |
| Cancer | Education | Illiteracy and primary school | 0.90 (0.75, 1.06) | Ref |
| Cancer | Education | High School and above | 1.10 (0.87, 1.38) | 0.165 |
| Cancer | Marriage | Single | 0.80 (0.61, 1.05) | Ref |
| Cancer | Marriage | Married | 1.02 (0.87, 1.20) | 0.129 |
| Cancer | Exercise | Never | 0.93 (0.77, 1.14) | Ref |
| Cancer | Exercise | 1–5 times/week | 1.06 (0.81, 1.39) | 0.451 |
| Cancer | Exercise | over 6 times/week | 0.93 (0.70, 1.23) | 0.966 |
| Cancer | Income | T1 | 0.75 (0.57, 0.98) | Ref |
| Cancer | Income | T2 | 1.03 (0.81, 1.31) | 0.083 |
| Cancer | Income | T3 | 1.11 (0.89, 1.38) | 0.024 |
| Cancer | Climate | Cold | 0.95 (0.72, 1.26) | Ref |
| Cancer | Climate | Moderate cold | 0.89 (0.67, 1.20) | 0.746 |
| Cancer | Climate | Moderate hot | 1.39 (0.84, 2.30) | 0.204 |
| Cancer | Climate | Hot | 0.93 (0.61, 1.41) | 0.921 |
| Reproductive diseases | Sex | Female | 1.07 (0.93, 1.24) | Ref |
| Reproductive diseases | Sex | Male | 1.04 (0.94, 1.16) | 0.756 |
| Reproductive diseases | Age | 60–74 yrs | 1.12 (1.01, 1.24) | Ref |
| Reproductive diseases | Age | over 75 yrs | 0.98 (0.84, 1.14) | 0.161 |
| Reproductive diseases | Urbanity | Urban | 1.12 (1.00, 1.27) | Ref |
| Reproductive diseases | Urbanity | Rural | 0.99 (0.87, 1.12) | 0.146 |
| Reproductive diseases | Education | Illiteracy and primary school | 1.09 (0.98, 1.21) | Ref |
| Reproductive diseases | Education | High School and above | 0.98 (0.84, 1.15) | 0.275 |
| Reproductive diseases | Marriage | Single | 1.06 (0.89, 1.27) | Ref |
| Reproductive diseases | Marriage | Married | 1.06 (0.96, 1.17) | 0.982 |
| Reproductive diseases | Exercise | Never | 1.10 (0.98, 1.24) | Ref |
| Reproductive diseases | Exercise | 1–5 times/week | 0.98 (0.82, 1.18) | 0.306 |
| Reproductive diseases | Exercise | over 6 times/week | 0.99 (0.83, 1.19) | 0.335 |
| Reproductive diseases | Income | T1 | 1.04 (0.90, 1.20) | Ref |
| Reproductive diseases | Income | T2 | 1.15 (0.98, 1.34) | 0.362 |
| Reproductive diseases | Income | T3 | 1.00 (0.86, 1.17) | 0.739 |
| Reproductive diseases | Climate | Cold | 0.84 (0.71, 0.98) | Ref |
| Reproductive diseases | Climate | Moderate cold | 1.54 (1.24, 1.92) | <0.001 |
| Reproductive diseases | Climate | Moderate hot | 1.43 (1.06, 1.93) | 0.002 |
| Reproductive diseases | Climate | Hot | 0.95 (0.71, 1.26) | 0.446 |

TV, temperature variability.

# Table S3. Risk (Odds ratio, OR) for all diseases associated with every 1℃ increase in TV during 2010–2014.

| Disease | Strata | Groups | Odds ratio (95% CI) | P for difference |
| --- | --- | --- | --- | --- |
| Cataract | Sex | Female | 1.04 (1.00, 1.08) | Ref |
| Cataract | Sex | Male | 1.03 (0.98, 1.08) | 0.624 |
| Cataract | Age | 60–74 yrs | 1.02 (0.98, 1.05) | Ref |
| Cataract | Age | over 75 yrs | 1.07 (1.02, 1.12) | 0.108 |
| Cataract | Urbanity | Urban | 1.05 (1.01, 1.10) | Ref |
| Cataract | Urbanity | Rural | 1.02 (0.98, 1.07) | 0.309 |
| Cataract | Education | Illiteracy and primary school | 1.05 (1.02, 1.09) | Ref |
| Cataract | Education | High School and above | 0.98 (0.92, 1.04) | 0.040 |
| Cataract | Marriage | Single | 1.05 (1.00, 1.10) | Ref |
| Cataract | Marriage | Married | 1.03 (0.99, 1.07) | 0.532 |
| Cataract | Exercise | Never | 1.04 (1.00, 1.09) | Ref |
| Cataract | Exercise | 1–5 times/week | 1.07 (1.01, 1.14) | 0.423 |
| Cataract | Exercise | over 6 times/week | 0.97 (0.91, 1.03) | 0.057 |
| Cataract | Income | T1 | 1.06 (1.01, 1.12) | Ref |
| Cataract | Income | T2 | 0.99 (0.94, 1.04) | 0.050 |
| Cataract | Income | T3 | 1.05 (0.99, 1.11) | 0.728 |
| Cataract | Climate | Cold | 0.94 (0.88, 1.00) | Ref |
| Cataract | Climate | Moderate cold | 1.04 (0.97, 1.11) | 0.039 |
| Cataract | Climate | Moderate hot | 1.26 (1.08, 1.48) | 0.001 |
| Cataract | Climate | Hot | 1.19 (1.07, 1.33) | <0.001 |
| Hypertension | Sex | Female | 1.02 (0.99, 1.05) | Ref |
| Hypertension | Sex | Male | 1.04 (1.01, 1.07) | 0.438 |
| Hypertension | Age | 60–74 yrs | 1.04 (1.01, 1.06) | Ref |
| Hypertension | Age | over 75 yrs | 1.01 (0.96, 1.05) | 0.234 |
| Hypertension | Urbanity | Urban | 1.03 (1.00, 1.06) | Ref |
| Hypertension | Urbanity | Rural | 1.02 (0.99, 1.05) | 0.592 |
| Hypertension | Education | Illiteracy and primary school | 1.03 (1.00, 1.05) | Ref |
| Hypertension | Education | High School and above | 1.03 (0.99, 1.07) | 0.907 |
| Hypertension | Marriage | Single | 1.01 (0.97, 1.05) | Ref |
| Hypertension | Marriage | Married | 1.04 (1.01, 1.07) | 0.165 |
| Hypertension | Exercise | Never | 1.04 (1.01, 1.07) | Ref |
| Hypertension | Exercise | 1–5 times/week | 1.02 (0.98, 1.06) | 0.524 |
| Hypertension | Exercise | over 6 times/week | 1.00 (0.95, 1.05) | 0.211 |
| Hypertension | Income | T1 | 1.02 (0.99, 1.06) | Ref |
| Hypertension | Income | T2 | 1.01 (0.98, 1.05) | 0.694 |
| Hypertension | Income | T3 | 1.05 (1.00, 1.09) | 0.475 |
| Hypertension | Climate | Cold | 0.93 (0.89, 0.98) | Ref |
| Hypertension | Climate | Moderate cold | 1.12 (1.07, 1.18) | <0.001 |
| Hypertension | Climate | Moderate hot | 1.17 (1.03, 1.32) | 0.001 |
| Hypertension | Climate | Hot | 1.13 (1.04, 1.23) | <0.001 |
| Diabetes | Sex | Female | 0.95 (0.91, 1.00) | Ref |
| Diabetes | Sex | Male | 1.03 (0.97, 1.10) | 0.031 |
| Diabetes | Age | 60–74 yrs | 1.00 (0.96, 1.04) | Ref |
| Diabetes | Age | over 75 yrs | 0.93 (0.87, 1.01) | 0.135 |
| Diabetes | Urbanity | Urban | 1.00 (0.96, 1.05) | Ref |
| Diabetes | Urbanity | Rural | 0.94 (0.88, 1.00) | 0.103 |
| Diabetes | Education | Illiteracy and primary school | 0.97 (0.92, 1.01) | Ref |
| Diabetes | Education | High School and above | 1.02 (0.95, 1.09) | 0.210 |
| Diabetes | Marriage | Single | 0.96 (0.89, 1.03) | Ref |
| Diabetes | Marriage | Married | 0.99 (0.95, 1.04) | 0.372 |
| Diabetes | Exercise | Never | 1.01 (0.95, 1.07) | Ref |
| Diabetes | Exercise | 1–5 times/week | 0.98 (0.91, 1.05) | 0.503 |
| Diabetes | Exercise | over 6 times/week | 0.94 (0.87, 1.01) | 0.122 |
| Diabetes | Income | T1 | 0.97 (0.90, 1.04) | Ref |
| Diabetes | Income | T2 | 0.93 (0.88, 1.00) | 0.493 |
| Diabetes | Income | T3 | 1.01 (0.95, 1.08) | 0.328 |
| Diabetes | Climate | Cold | 0.97 (0.90, 1.05) | Ref |
| Diabetes | Climate | Moderate cold | 0.89 (0.82, 0.97) | 0.109 |
| Diabetes | Climate | Moderate hot | 1.31 (1.05, 1.63) | 0.011 |
| Diabetes | Climate | Hot | 1.21 (1.03, 1.41) | 0.016 |
| Cardio-cerebrovascular diseases | Sex | Female | 1.12 (1.08, 1.15) | Ref |
| Cardio-cerebrovascular diseases | Sex | Male | 1.08 (1.04, 1.12) | 0.206 |
| Cardio-cerebrovascular diseases | Age | 60–74 yrs | 1.11 (1.08, 1.14) | Ref |
| Cardio-cerebrovascular diseases | Age | over 75 yrs | 1.07 (1.02, 1.12) | 0.154 |
| Cardio-cerebrovascular diseases | Urbanity | Urban | 1.13 (1.10, 1.17) | Ref |
| Cardio-cerebrovascular diseases | Urbanity | Rural | 1.06 (1.02, 1.10) | 0.011 |
| Cardio-cerebrovascular diseases | Education | Illiteracy and primary school | 1.10 (1.06, 1.13) | Ref |
| Cardio-cerebrovascular diseases | Education | High School and above | 1.11 (1.06, 1.17) | 0.570 |
| Cardio-cerebrovascular diseases | Marriage | Single | 1.10 (1.05, 1.15) | Ref |
| Cardio-cerebrovascular diseases | Marriage | Married | 1.10 (1.07, 1.14) | 0.849 |
| Cardio-cerebrovascular diseases | Exercise | Never | 1.08 (1.04, 1.12) | Ref |
| Cardio-cerebrovascular diseases | Exercise | 1–5 times/week | 1.14 (1.09, 1.20) | 0.058 |
| Cardio-cerebrovascular diseases | Exercise | over 6 times/week | 1.08 (1.02, 1.14) | 0.976 |
| Cardio-cerebrovascular diseases | Income | T1 | 1.08 (1.04, 1.13) | Ref |
| Cardio-cerebrovascular diseases | Income | T2 | 1.09 (1.05, 1.14) | 0.759 |
| Cardio-cerebrovascular diseases | Income | T3 | 1.13 (1.08, 1.18) | 0.181 |
| Cardio-cerebrovascular diseases | Climate | Cold | 1.06 (1.01, 1.11) | Ref |
| Cardio-cerebrovascular diseases | Climate | Moderate cold | 1.19 (1.13, 1.25) | 0.002 |
| Cardio-cerebrovascular diseases | Climate | Moderate hot | 1.33 (1.16, 1.54) | 0.003 |
| Cardio-cerebrovascular diseases | Climate | Hot | 1.26 (1.14, 1.39) | 0.003 |
| Stomach diseases | Sex | Female | 1.09 (1.05, 1.13) | Ref |
| Stomach diseases | Sex | Male | 1.04 (1.00, 1.09) | 0.083 |
| Stomach diseases | Age | 60–74 yrs | 1.08 (1.05, 1.11) | Ref |
| Stomach diseases | Age | over 75 yrs | 1.05 (0.99, 1.11) | 0.421 |
| Stomach diseases | Urbanity | Urban | 1.08 (1.03, 1.12) | Ref |
| Stomach diseases | Urbanity | Rural | 1.06 (1.02, 1.10) | 0.641 |
| Stomach diseases | Education | Illiteracy and primary school | 1.09 (1.06, 1.13) | Ref |
| Stomach diseases | Education | High School and above | 1.02 (0.96, 1.08) | 0.039 |
| Stomach diseases | Marriage | Single | 1.11 (1.05, 1.16) | Ref |
| Stomach diseases | Marriage | Married | 1.06 (1.03, 1.09) | 0.174 |
| Stomach diseases | Exercise | Never | 1.06 (1.02, 1.10) | Ref |
| Stomach diseases | Exercise | 1–5 times/week | 1.07 (1.01, 1.13) | 0.842 |
| Stomach diseases | Exercise | over 6 times/week | 1.09 (1.02, 1.16) | 0.540 |
| Stomach diseases | Income | T1 | 1.13 (1.08, 1.18) | Ref |
| Stomach diseases | Income | T2 | 1.07 (1.02, 1.12) | 0.089 |
| Stomach diseases | Income | T3 | 1.01 (0.95, 1.07) | 0.003 |
| Stomach diseases | Climate | Cold | 0.99 (0.93, 1.05) | Ref |
| Stomach diseases | Climate | Moderate cold | 1.23 (1.16, 1.32) | <0.001 |
| Stomach diseases | Climate | Moderate hot | 1.43 (1.23, 1.66) | <0.001 |
| Stomach diseases | Climate | Hot | 1.18 (1.06, 1.31) | 0.004 |
| Arthritis | Sex | Female | 1.04 (1.01, 1.07) | Ref |
| Arthritis | Sex | Male | 1.03 (1.00, 1.06) | 0.592 |
| Arthritis | Age | 60–74 yrs | 1.05 (1.03, 1.08) | Ref |
| Arthritis | Age | over 75 yrs | 0.98 (0.94, 1.02) | 0.005 |
| Arthritis | Urbanity | Urban | 1.03 (1.00, 1.06) | Ref |
| Arthritis | Urbanity | Rural | 1.02 (0.99, 1.05) | 0.690 |
| Arthritis | Education | Illiteracy and primary school | 1.04 (1.01, 1.06) | Ref |
| Arthritis | Education | High School and above | 1.02 (0.98, 1.06) | 0.531 |
| Arthritis | Marriage | Single | 0.99 (0.95, 1.03) | Ref |
| Arthritis | Marriage | Married | 1.05 (1.02, 1.08) | 0.011 |
| Arthritis | Exercise | Never | 0.99 (0.96, 1.02) | Ref |
| Arthritis | Exercise | 1–5 times/week | 1.08 (1.04, 1.12) | 0.001 |
| Arthritis | Exercise | over 6 times/week | 1.04 (0.99, 1.09) | 0.150 |
| Arthritis | Income | T1 | 1.06 (1.02, 1.10) | Ref |
| Arthritis | Income | T2 | 1.03 (0.99, 1.07) | 0.277 |
| Arthritis | Income | T3 | 0.98 (0.94, 1.02) | 0.003 |
| Arthritis | Climate | Cold | 1.05 (1.00, 1.10) | Ref |
| Arthritis | Climate | Moderate cold | 1.14 (1.08, 1.19) | 0.019 |
| Arthritis | Climate | Moderate hot | 1.33 (1.17, 1.50) | <0.001 |
| Arthritis | Climate | Hot | 1.14 (1.05, 1.23) | 0.082 |
| Chronic lung diseases | Sex | Female | 1.07 (1.01, 1.12) | Ref |
| Chronic lung diseases | Sex | Male | 1.04 (0.99, 1.09) | 0.480 |
| Chronic lung diseases | Age | 60–74 yrs | 1.04 (1.00, 1.09) | Ref |
| Chronic lung diseases | Age | over 75 yrs | 1.06 (1.00, 1.13) | 0.638 |
| Chronic lung diseases | Urbanity | Urban | 1.05 (1.00, 1.10) | Ref |
| Chronic lung diseases | Urbanity | Rural | 1.05 (1.00, 1.10) | 0.904 |
| Chronic lung diseases | Education | Illiteracy and primary school | 1.05 (1.01, 1.10) | Ref |
| Chronic lung diseases | Education | High School and above | 1.05 (0.97, 1.13) | 0.960 |
| Chronic lung diseases | Marriage | Single | 1.01 (0.95, 1.07) | Ref |
| Chronic lung diseases | Marriage | Married | 1.07 (1.03, 1.12) | 0.116 |
| Chronic lung diseases | Exercise | Never | 1.05 (1.01, 1.10) | Ref |
| Chronic lung diseases | Exercise | 1–5 times/week | 1.10 (1.03, 1.19) | 0.271 |
| Chronic lung diseases | Exercise | over 6 times/week | 0.98 (0.90, 1.07) | 0.174 |
| Chronic lung diseases | Income | T1 | 1.03 (0.98, 1.09) | Ref |
| Chronic lung diseases | Income | T2 | 1.05 (0.99, 1.11) | 0.764 |
| Chronic lung diseases | Income | T3 | 1.08 (1.00, 1.16) | 0.410 |
| Chronic lung diseases | Climate | Cold | 0.92 (0.85, 0.99) | Ref |
| Chronic lung diseases | Climate | Moderate cold | 1.23 (1.13, 1.34) | <0.001 |
| Chronic lung diseases | Climate | Moderate hot | 1.12 (0.93, 1.36) | 0.055 |
| Chronic lung diseases | Climate | Hot | 1.02 (0.90, 1.16) | 0.140 |
| Asthma | Sex | Female | 1.06 (0.99, 1.14) | Ref |
| Asthma | Sex | Male | 1.14 (1.06, 1.23) | 0.131 |
| Asthma | Age | 60–74 yrs | 1.11 (1.05, 1.18) | Ref |
| Asthma | Age | over 75 yrs | 1.08 (0.99, 1.17) | 0.537 |
| Asthma | Urbanity | Urban | 1.06 (0.98, 1.15) | Ref |
| Asthma | Urbanity | Rural | 1.12 (1.05, 1.20) | 0.320 |
| Asthma | Education | Illiteracy and primary school | 1.11 (1.05, 1.18) | Ref |
| Asthma | Education | High School and above | 1.05 (0.93, 1.19) | 0.396 |
| Asthma | Marriage | Single | 1.07 (0.98, 1.17) | Ref |
| Asthma | Marriage | Married | 1.11 (1.05, 1.18) | 0.499 |
| Asthma | Exercise | Never | 1.06 (1.00, 1.13) | Ref |
| Asthma | Exercise | 1–5 times/week | 1.17 (1.05, 1.30) | 0.134 |
| Asthma | Exercise | over 6 times/week | 1.15 (1.00, 1.32) | 0.330 |
| Asthma | Income | T1 | 1.06 (0.99, 1.15) | Ref |
| Asthma | Income | T2 | 1.16 (1.06, 1.27) | 0.152 |
| Asthma | Income | T3 | 1.08 (0.96, 1.21) | 0.869 |
| Asthma | Climate | Cold | 0.92 (0.83, 1.03) | Ref |
| Asthma | Climate | Moderate cold | 1.13 (1.00, 1.27) | 0.016 |
| Asthma | Climate | Moderate hot | 1.42 (1.05, 1.92) | 0.008 |
| Asthma | Climate | Hot | 1.00 (0.83, 1.21) | 0.486 |
| Cancer | Sex | Female | 1.04 (0.90, 1.20) | Ref |
| Cancer | Sex | Male | 0.93 (0.79, 1.08) | 0.281 |
| Cancer | Age | 60–74 yrs | 1.00 (0.89, 1.13) | Ref |
| Cancer | Age | over 75 yrs | 0.88 (0.70, 1.11) | 0.330 |
| Cancer | Urbanity | Urban | 1.03 (0.90, 1.18) | Ref |
| Cancer | Urbanity | Rural | 0.93 (0.78, 1.10) | 0.352 |
| Cancer | Education | Illiteracy and primary school | 0.93 (0.82, 1.06) | Ref |
| Cancer | Education | High School and above | 1.09 (0.91, 1.31) | 0.162 |
| Cancer | Marriage | Single | 0.89 (0.73, 1.09) | Ref |
| Cancer | Marriage | Married | 1.02 (0.90, 1.15) | 0.256 |
| Cancer | Exercise | Never | 0.95 (0.82, 1.10) | Ref |
| Cancer | Exercise | 1–5 times/week | 1.11 (0.90, 1.37) | 0.219 |
| Cancer | Exercise | over 6 times/week | 0.97 (0.78, 1.21) | 0.879 |
| Cancer | Income | T1 | 0.89 (0.72, 1.10) | Ref |
| Cancer | Income | T2 | 0.99 (0.82, 1.19) | 0.454 |
| Cancer | Income | T3 | 1.10 (0.93, 1.30) | 0.114 |
| Cancer | Climate | Cold | 1.01 (0.80, 1.26) | Ref |
| Cancer | Climate | Moderate cold | 1.16 (0.91, 1.47) | 0.413 |
| Cancer | Climate | Moderate hot | 1.36 (0.82, 2.25) | 0.287 |
| Cancer | Climate | Hot | 0.87 (0.59, 1.29) | 0.531 |
| Reproductive diseases | Sex | Female | 1.07 (0.96, 1.20) | Ref |
| Reproductive diseases | Sex | Male | 1.02 (0.94, 1.11) | 0.494 |
| Reproductive diseases | Age | 60–74 yrs | 1.09 (1.01, 1.19) | Ref |
| Reproductive diseases | Age | over 75 yrs | 0.96 (0.85, 1.07) | 0.058 |
| Reproductive diseases | Urbanity | Urban | 1.07 (0.97, 1.17) | Ref |
| Reproductive diseases | Urbanity | Rural | 1.01 (0.92, 1.12) | 0.455 |
| Reproductive diseases | Education | Illiteracy and primary school | 1.06 (0.98, 1.15) | Ref |
| Reproductive diseases | Education | High School and above | 0.99 (0.88, 1.12) | 0.374 |
| Reproductive diseases | Marriage | Single | 1.06 (0.93, 1.22) | Ref |
| Reproductive diseases | Marriage | Married | 1.03 (0.96, 1.11) | 0.687 |
| Reproductive diseases | Exercise | Never | 1.10 (1.01, 1.21) | Ref |
| Reproductive diseases | Exercise | 1–5 times/week | 0.98 (0.85, 1.13) | 0.178 |
| Reproductive diseases | Exercise | over 6 times/week | 0.94 (0.82, 1.08) | 0.060 |
| Reproductive diseases | Income | T1 | 1.08 (0.96, 1.20) | Ref |
| Reproductive diseases | Income | T2 | 1.12 (0.99, 1.26) | 0.639 |
| Reproductive diseases | Income | T3 | 0.97 (0.86, 1.09) | 0.195 |
| Reproductive diseases | Climate | Cold | 0.85 (0.75, 0.97) | Ref |
| Reproductive diseases | Climate | Moderate cold | 1.40 (1.18, 1.67) | <0.001 |
| Reproductive diseases | Climate | Moderate hot | 1.54 (1.15, 2.07) | <0.001 |
| Reproductive diseases | Climate | Hot | 1.03 (0.79, 1.35) | 0.210 |

TV, temperature variability.

# Table S4. Risk (Odds ratio, OR) for all diseases associated with every 1℃ increase in TV during 2011–2014.

| Disease | Strata | Groups | Odds ratio (95% CI) | P for difference |
| --- | --- | --- | --- | --- |
| Cataract | Sex | Female | 1.04 (1.01, 1.08) | Ref |
| Cataract | Sex | Male | 1.03 (0.98, 1.08) | 0.602 |
| Cataract | Age | 60–74 yrs | 1.02 (0.98, 1.06) | Ref |
| Cataract | Age | over 75 yrs | 1.07 (1.02, 1.12) | 0.103 |
| Cataract | Urbanity | Urban | 1.05 (1.01, 1.09) | Ref |
| Cataract | Urbanity | Rural | 1.03 (0.98, 1.07) | 0.408 |
| Cataract | Education | Illiteracy and primary school | 1.05 (1.02, 1.09) | Ref |
| Cataract | Education | High School and above | 0.98 (0.92, 1.04) | 0.035 |
| Cataract | Marriage | Single | 1.05 (1.00, 1.10) | Ref |
| Cataract | Marriage | Married | 1.03 (0.99, 1.07) | 0.496 |
| Cataract | Exercise | Never | 1.05 (1.01, 1.09) | Ref |
| Cataract | Exercise | 1–5 times/week | 1.07 (1.01, 1.14) | 0.487 |
| Cataract | Exercise | over 6 times/week | 0.97 (0.91, 1.03) | 0.045 |
| Cataract | Income | T1 | 1.07 (1.02, 1.12) | Ref |
| Cataract | Income | T2 | 1.00 (0.95, 1.05) | 0.054 |
| Cataract | Income | T3 | 1.05 (1.00, 1.11) | 0.736 |
| Cataract | Climate | Cold | 0.94 (0.89, 1.00) | Ref |
| Cataract | Climate | Moderate cold | 1.05 (0.98, 1.12) | 0.029 |
| Cataract | Climate | Moderate hot | 1.28 (1.10, 1.49) | <0.001 |
| Cataract | Climate | Hot | 1.18 (1.06, 1.32) | <0.001 |
| Hypertension | Sex | Female | 1.02 (1.00, 1.05) | Ref |
| Hypertension | Sex | Male | 1.04 (1.01, 1.08) | 0.448 |
| Hypertension | Age | 60–74 yrs | 1.04 (1.01, 1.06) | Ref |
| Hypertension | Age | over 75 yrs | 1.01 (0.97, 1.05) | 0.236 |
| Hypertension | Urbanity | Urban | 1.04 (1.01, 1.07) | Ref |
| Hypertension | Urbanity | Rural | 1.02 (0.99, 1.05) | 0.457 |
| Hypertension | Education | Illiteracy and primary school | 1.03 (1.00, 1.05) | Ref |
| Hypertension | Education | High School and above | 1.04 (0.99, 1.08) | 0.753 |
| Hypertension | Marriage | Single | 1.01 (0.97, 1.05) | Ref |
| Hypertension | Marriage | Married | 1.04 (1.02, 1.07) | 0.175 |
| Hypertension | Exercise | Never | 1.04 (1.01, 1.07) | Ref |
| Hypertension | Exercise | 1–5 times/week | 1.02 (0.98, 1.06) | 0.486 |
| Hypertension | Exercise | over 6 times/week | 1.01 (0.96, 1.06) | 0.288 |
| Hypertension | Income | T1 | 1.02 (0.99, 1.06) | Ref |
| Hypertension | Income | T2 | 1.02 (0.98, 1.06) | 0.770 |
| Hypertension | Income | T3 | 1.05 (1.01, 1.10) | 0.345 |
| Hypertension | Climate | Cold | 0.94 (0.90, 0.98) | Ref |
| Hypertension | Climate | Moderate cold | 1.11 (1.06, 1.16) | <0.001 |
| Hypertension | Climate | Moderate hot | 1.18 (1.05, 1.32) | 0.001 |
| Hypertension | Climate | Hot | 1.16 (1.07, 1.27) | <0.001 |
| Diabetes | Sex | Female | 0.95 (0.91, 1.00) | Ref |
| Diabetes | Sex | Male | 1.04 (0.98, 1.10) | 0.019 |
| Diabetes | Age | 60–74 yrs | 1.00 (0.96, 1.05) | Ref |
| Diabetes | Age | over 75 yrs | 0.94 (0.87, 1.01) | 0.110 |
| Diabetes | Urbanity | Urban | 1.01 (0.96, 1.05) | Ref |
| Diabetes | Urbanity | Rural | 0.94 (0.88, 1.00) | 0.083 |
| Diabetes | Education | Illiteracy and primary school | 0.97 (0.93, 1.01) | Ref |
| Diabetes | Education | High School and above | 1.02 (0.96, 1.09) | 0.176 |
| Diabetes | Marriage | Single | 0.96 (0.90, 1.03) | Ref |
| Diabetes | Marriage | Married | 1.00 (0.96, 1.04) | 0.362 |
| Diabetes | Exercise | Never | 1.01 (0.95, 1.07) | Ref |
| Diabetes | Exercise | 1–5 times/week | 0.98 (0.92, 1.05) | 0.559 |
| Diabetes | Exercise | over 6 times/week | 0.95 (0.88, 1.01) | 0.155 |
| Diabetes | Income | T1 | 0.96 (0.90, 1.03) | Ref |
| Diabetes | Income | T2 | 0.94 (0.88, 1.00) | 0.610 |
| Diabetes | Income | T3 | 1.02 (0.96, 1.09) | 0.201 |
| Diabetes | Climate | Cold | 0.98 (0.91, 1.05) | Ref |
| Diabetes | Climate | Moderate cold | 0.89 (0.82, 0.96) | 0.082 |
| Diabetes | Climate | Moderate hot | 1.37 (1.11, 1.68) | 0.003 |
| Diabetes | Climate | Hot | 1.24 (1.06, 1.45) | 0.008 |
| Cardio-cerebrovascular diseases | Sex | Female | 1.11 (1.08, 1.15) | Ref |
| Cardio-cerebrovascular diseases | Sex | Male | 1.08 (1.04, 1.12) | 0.239 |
| Cardio-cerebrovascular diseases | Age | 60–74 yrs | 1.11 (1.08, 1.14) | Ref |
| Cardio-cerebrovascular diseases | Age | over 75 yrs | 1.07 (1.02, 1.12) | 0.136 |
| Cardio-cerebrovascular diseases | Urbanity | Urban | 1.13 (1.10, 1.17) | Ref |
| Cardio-cerebrovascular diseases | Urbanity | Rural | 1.06 (1.02, 1.10) | 0.012 |
| Cardio-cerebrovascular diseases | Education | Illiteracy and primary school | 1.09 (1.06, 1.13) | Ref |
| Cardio-cerebrovascular diseases | Education | High School and above | 1.11 (1.06, 1.17) | 0.553 |
| Cardio-cerebrovascular diseases | Marriage | Single | 1.10 (1.05, 1.15) | Ref |
| Cardio-cerebrovascular diseases | Marriage | Married | 1.10 (1.07, 1.13) | 0.956 |
| Cardio-cerebrovascular diseases | Exercise | Never | 1.08 (1.04, 1.12) | Ref |
| Cardio-cerebrovascular diseases | Exercise | 1–5 times/week | 1.14 (1.09, 1.19) | 0.062 |
| Cardio-cerebrovascular diseases | Exercise | over 6 times/week | 1.09 (1.03, 1.15) | 0.741 |
| Cardio-cerebrovascular diseases | Income | T1 | 1.08 (1.03, 1.12) | Ref |
| Cardio-cerebrovascular diseases | Income | T2 | 1.09 (1.05, 1.14) | 0.634 |
| Cardio-cerebrovascular diseases | Income | T3 | 1.13 (1.08, 1.18) | 0.131 |
| Cardio-cerebrovascular diseases | Climate | Cold | 1.06 (1.01, 1.11) | Ref |
| Cardio-cerebrovascular diseases | Climate | Moderate cold | 1.19 (1.13, 1.26) | 0.001 |
| Cardio-cerebrovascular diseases | Climate | Moderate hot | 1.36 (1.19, 1.56) | 0.001 |
| Cardio-cerebrovascular diseases | Climate | Hot | 1.25 (1.13, 1.38) | 0.003 |
| Stomach diseases | Sex | Female | 1.09 (1.05, 1.13) | Ref |
| Stomach diseases | Sex | Male | 1.04 (1.00, 1.08) | 0.085 |
| Stomach diseases | Age | 60–74 yrs | 1.07 (1.04, 1.11) | Ref |
| Stomach diseases | Age | over 75 yrs | 1.05 (0.99, 1.11) | 0.481 |
| Stomach diseases | Urbanity | Urban | 1.07 (1.03, 1.11) | Ref |
| Stomach diseases | Urbanity | Rural | 1.06 (1.02, 1.10) | 0.751 |
| Stomach diseases | Education | Illiteracy and primary school | 1.09 (1.05, 1.12) | Ref |
| Stomach diseases | Education | High School and above | 1.01 (0.96, 1.07) | 0.039 |
| Stomach diseases | Marriage | Single | 1.10 (1.05, 1.16) | Ref |
| Stomach diseases | Marriage | Married | 1.05 (1.02, 1.09) | 0.173 |
| Stomach diseases | Exercise | Never | 1.05 (1.02, 1.09) | Ref |
| Stomach diseases | Exercise | 1–5 times/week | 1.07 (1.01, 1.12) | 0.736 |
| Stomach diseases | Exercise | over 6 times/week | 1.08 (1.02, 1.16) | 0.452 |
| Stomach diseases | Income | T1 | 1.12 (1.08, 1.17) | Ref |
| Stomach diseases | Income | T2 | 1.06 (1.01, 1.11) | 0.071 |
| Stomach diseases | Income | T3 | 1.01 (0.95, 1.06) | 0.002 |
| Stomach diseases | Climate | Cold | 0.98 (0.92, 1.03) | Ref |
| Stomach diseases | Climate | Moderate cold | 1.22 (1.14, 1.30) | <0.001 |
| Stomach diseases | Climate | Moderate hot | 1.36 (1.17, 1.56) | <0.001 |
| Stomach diseases | Climate | Hot | 1.19 (1.07, 1.32) | 0.001 |
| Arthritis | Sex | Female | 1.04 (1.01, 1.06) | Ref |
| Arthritis | Sex | Male | 1.02 (0.99, 1.06) | 0.627 |
| Arthritis | Age | 60–74 yrs | 1.05 (1.02, 1.07) | Ref |
| Arthritis | Age | over 75 yrs | 0.98 (0.94, 1.02) | 0.008 |
| Arthritis | Urbanity | Urban | 1.02 (0.99, 1.05) | Ref |
| Arthritis | Urbanity | Rural | 1.01 (0.98, 1.05) | 0.723 |
| Arthritis | Education | Illiteracy and primary school | 1.03 (1.01, 1.06) | Ref |
| Arthritis | Education | High School and above | 1.02 (0.98, 1.06) | 0.523 |
| Arthritis | Marriage | Single | 0.99 (0.95, 1.03) | Ref |
| Arthritis | Marriage | Married | 1.05 (1.02, 1.07) | 0.015 |
| Arthritis | Exercise | Never | 0.99 (0.96, 1.02) | Ref |
| Arthritis | Exercise | 1–5 times/week | 1.07 (1.03, 1.12) | 0.001 |
| Arthritis | Exercise | over 6 times/week | 1.04 (0.99, 1.09) | 0.091 |
| Arthritis | Income | T1 | 1.05 (1.02, 1.09) | Ref |
| Arthritis | Income | T2 | 1.03 (0.99, 1.07) | 0.381 |
| Arthritis | Income | T3 | 0.98 (0.94, 1.02) | 0.008 |
| Arthritis | Climate | Cold | 1.03 (0.99, 1.08) | Ref |
| Arthritis | Climate | Moderate cold | 1.12 (1.07, 1.17) | 0.017 |
| Arthritis | Climate | Moderate hot | 1.28 (1.14, 1.44) | 0.001 |
| Arthritis | Climate | Hot | 1.16 (1.07, 1.25) | 0.011 |
| Chronic lung diseases | Sex | Female | 1.07 (1.01, 1.12) | Ref |
| Chronic lung diseases | Sex | Male | 1.04 (0.99, 1.09) | 0.453 |
| Chronic lung diseases | Age | 60–74 yrs | 1.04 (1.00, 1.09) | Ref |
| Chronic lung diseases | Age | over 75 yrs | 1.06 (0.99, 1.13) | 0.711 |
| Chronic lung diseases | Urbanity | Urban | 1.05 (0.99, 1.10) | Ref |
| Chronic lung diseases | Urbanity | Rural | 1.05 (1.00, 1.10) | 0.898 |
| Chronic lung diseases | Education | Illiteracy and primary school | 1.05 (1.01, 1.09) | Ref |
| Chronic lung diseases | Education | High School and above | 1.05 (0.97, 1.13) | 0.934 |
| Chronic lung diseases | Marriage | Single | 1.01 (0.95, 1.07) | Ref |
| Chronic lung diseases | Marriage | Married | 1.07 (1.03, 1.12) | 0.120 |
| Chronic lung diseases | Exercise | Never | 1.05 (1.00, 1.10) | Ref |
| Chronic lung diseases | Exercise | 1–5 times/week | 1.10 (1.03, 1.18) | 0.241 |
| Chronic lung diseases | Exercise | over 6 times/week | 0.99 (0.91, 1.07) | 0.206 |
| Chronic lung diseases | Income | T1 | 1.03 (0.98, 1.09) | Ref |
| Chronic lung diseases | Income | T2 | 1.05 (0.99, 1.11) | 0.674 |
| Chronic lung diseases | Income | T3 | 1.08 (1.00, 1.16) | 0.343 |
| Chronic lung diseases | Climate | Cold | 0.92 (0.85, 0.98) | Ref |
| Chronic lung diseases | Climate | Moderate cold | 1.22 (1.12, 1.33) | <0.001 |
| Chronic lung diseases | Climate | Moderate hot | 1.10 (0.91, 1.32) | 0.069 |
| Chronic lung diseases | Climate | Hot | 1.03 (0.91, 1.18) | 0.099 |
| Asthma | Sex | Female | 1.06 (0.99, 1.13) | Ref |
| Asthma | Sex | Male | 1.14 (1.06, 1.23) | 0.123 |
| Asthma | Age | 60–74 yrs | 1.11 (1.04, 1.18) | Ref |
| Asthma | Age | over 75 yrs | 1.08 (0.99, 1.17) | 0.548 |
| Asthma | Urbanity | Urban | 1.06 (0.98, 1.14) | Ref |
| Asthma | Urbanity | Rural | 1.12 (1.05, 1.20) | 0.249 |
| Asthma | Education | Illiteracy and primary school | 1.11 (1.05, 1.18) | Ref |
| Asthma | Education | High School and above | 1.05 (0.93, 1.18) | 0.387 |
| Asthma | Marriage | Single | 1.07 (0.98, 1.17) | Ref |
| Asthma | Marriage | Married | 1.11 (1.04, 1.18) | 0.509 |
| Asthma | Exercise | Never | 1.06 (1.00, 1.13) | Ref |
| Asthma | Exercise | 1–5 times/week | 1.17 (1.05, 1.29) | 0.117 |
| Asthma | Exercise | over 6 times/week | 1.14 (0.99, 1.31) | 0.345 |
| Asthma | Income | T1 | 1.06 (0.98, 1.14) | Ref |
| Asthma | Income | T2 | 1.16 (1.06, 1.27) | 0.111 |
| Asthma | Income | T3 | 1.08 (0.96, 1.20) | 0.810 |
| Asthma | Climate | Cold | 0.92 (0.82, 1.02) | Ref |
| Asthma | Climate | Moderate cold | 1.12 (1.00, 1.26) | 0.013 |
| Asthma | Climate | Moderate hot | 1.37 (1.03, 1.82) | 0.010 |
| Asthma | Climate | Hot | 1.02 (0.84, 1.23) | 0.350 |
| Cancer | Sex | Female | 1.04 (0.90, 1.19) | Ref |
| Cancer | Sex | Male | 0.93 (0.80, 1.08) | 0.286 |
| Cancer | Age | 60–74 yrs | 1.00 (0.89, 1.13) | Ref |
| Cancer | Age | over 75 yrs | 0.88 (0.71, 1.11) | 0.322 |
| Cancer | Urbanity | Urban | 1.03 (0.90, 1.17) | Ref |
| Cancer | Urbanity | Rural | 0.92 (0.78, 1.09) | 0.325 |
| Cancer | Education | Illiteracy and primary school | 0.93 (0.82, 1.06) | Ref |
| Cancer | Education | High School and above | 1.10 (0.92, 1.31) | 0.141 |
| Cancer | Marriage | Single | 0.89 (0.73, 1.09) | Ref |
| Cancer | Marriage | Married | 1.02 (0.90, 1.15) | 0.252 |
| Cancer | Exercise | Never | 0.94 (0.82, 1.09) | Ref |
| Cancer | Exercise | 1–5 times/week | 1.10 (0.90, 1.35) | 0.217 |
| Cancer | Exercise | over 6 times/week | 0.98 (0.79, 1.22) | 0.775 |
| Cancer | Income | T1 | 0.88 (0.72, 1.08) | Ref |
| Cancer | Income | T2 | 0.99 (0.82, 1.18) | 0.412 |
| Cancer | Income | T3 | 1.11 (0.94, 1.30) | 0.084 |
| Cancer | Climate | Cold | 0.99 (0.79, 1.23) | Ref |
| Cancer | Climate | Moderate cold | 1.14 (0.90, 1.44) | 0.386 |
| Cancer | Climate | Moderate hot | 1.38 (0.85, 2.22) | 0.213 |
| Cancer | Climate | Hot | 0.88 (0.60, 1.30) | 0.629 |
| Reproductive diseases | Sex | Female | 1.07 (0.96, 1.19) | Ref |
| Reproductive diseases | Sex | Male | 1.02 (0.94, 1.10) | 0.455 |
| Reproductive diseases | Age | 60–74 yrs | 1.09 (1.01, 1.18) | Ref |
| Reproductive diseases | Age | over 75 yrs | 0.96 (0.86, 1.07) | 0.072 |
| Reproductive diseases | Urbanity | Urban | 1.06 (0.97, 1.16) | Ref |
| Reproductive diseases | Urbanity | Rural | 1.01 (0.92, 1.12) | 0.518 |
| Reproductive diseases | Education | Illiteracy and primary school | 1.05 (0.97, 1.14) | Ref |
| Reproductive diseases | Education | High School and above | 1.00 (0.88, 1.12) | 0.428 |
| Reproductive diseases | Marriage | Single | 1.05 (0.92, 1.21) | Ref |
| Reproductive diseases | Marriage | Married | 1.03 (0.96, 1.11) | 0.750 |
| Reproductive diseases | Exercise | Never | 1.10 (1.00, 1.20) | Ref |
| Reproductive diseases | Exercise | 1–5 times/week | 0.98 (0.85, 1.12) | 0.167 |
| Reproductive diseases | Exercise | over 6 times/week | 0.95 (0.83, 1.09) | 0.083 |
| Reproductive diseases | Income | T1 | 1.07 (0.95, 1.19) | Ref |
| Reproductive diseases | Income | T2 | 1.12 (0.99, 1.25) | 0.590 |
| Reproductive diseases | Income | T3 | 0.97 (0.86, 1.08) | 0.221 |
| Reproductive diseases | Climate | Cold | 0.83 (0.73, 0.95) | Ref |
| Reproductive diseases | Climate | Moderate cold | 1.41 (1.19, 1.68) | <0.001 |
| Reproductive diseases | Climate | Moderate hot | 1.52 (1.15, 2.01) | <0.001 |
| Reproductive diseases | Climate | Hot | 1.03 (0.79, 1.35) | 0.167 |

TV, temperature variability.

# Table S5. Risk (Odds ratio, OR) for all diseases associated with every 1℃ increase in TV during 2012–2014.

| Disease | Strata | Groups | Odds ratio (95% CI) | P for difference |
| --- | --- | --- | --- | --- |
| Cataract | Sex | Female | 1.05 (1.02, 1.09) | Ref |
| Cataract | Sex | Male | 1.03 (0.98, 1.08) | 0.464 |
| Cataract | Age | 60–74 yrs | 1.03 (0.99, 1.07) | Ref |
| Cataract | Age | over 75 yrs | 1.08 (1.03, 1.13) | 0.118 |
| Cataract | Urbanity | Urban | 1.06 (1.02, 1.11) | Ref |
| Cataract | Urbanity | Rural | 1.03 (0.99, 1.08) | 0.301 |
| Cataract | Education | Illiteracy and primary school | 1.06 (1.03, 1.10) | Ref |
| Cataract | Education | High School and above | 0.98 (0.92, 1.04) | 0.018 |
| Cataract | Marriage | Single | 1.06 (1.01, 1.11) | Ref |
| Cataract | Marriage | Married | 1.04 (1.00, 1.08) | 0.463 |
| Cataract | Exercise | Never | 1.05 (1.01, 1.10) | Ref |
| Cataract | Exercise | 1–5 times/week | 1.09 (1.03, 1.15) | 0.350 |
| Cataract | Exercise | over 6 times/week | 0.97 (0.91, 1.04) | 0.046 |
| Cataract | Income | T1 | 1.07 (1.02, 1.13) | Ref |
| Cataract | Income | T2 | 1.00 (0.95, 1.06) | 0.066 |
| Cataract | Income | T3 | 1.06 (1.00, 1.12) | 0.760 |
| Cataract | Climate | Cold | 0.94 (0.89, 1.00) | Ref |
| Cataract | Climate | Moderate cold | 1.05 (0.98, 1.13) | 0.018 |
| Cataract | Climate | Moderate hot | 1.26 (1.10, 1.46) | <0.001 |
| Cataract | Climate | Hot | 1.29 (1.16, 1.45) | <0.001 |
| Hypertension | Sex | Female | 1.03 (1.00, 1.06) | Ref |
| Hypertension | Sex | Male | 1.04 (1.01, 1.08) | 0.476 |
| Hypertension | Age | 60–74 yrs | 1.04 (1.02, 1.07) | Ref |
| Hypertension | Age | over 75 yrs | 1.01 (0.97, 1.05) | 0.181 |
| Hypertension | Urbanity | Urban | 1.04 (1.01, 1.07) | Ref |
| Hypertension | Urbanity | Rural | 1.02 (0.99, 1.05) | 0.333 |
| Hypertension | Education | Illiteracy and primary school | 1.03 (1.01, 1.06) | Ref |
| Hypertension | Education | High School and above | 1.04 (0.99, 1.08) | 0.851 |
| Hypertension | Marriage | Single | 1.01 (0.97, 1.05) | Ref |
| Hypertension | Marriage | Married | 1.05 (1.02, 1.07) | 0.148 |
| Hypertension | Exercise | Never | 1.04 (1.01, 1.08) | Ref |
| Hypertension | Exercise | 1–5 times/week | 1.02 (0.98, 1.06) | 0.367 |
| Hypertension | Exercise | over 6 times/week | 1.01 (0.96, 1.06) | 0.241 |
| Hypertension | Income | T1 | 1.03 (0.99, 1.07) | Ref |
| Hypertension | Income | T2 | 1.02 (0.98, 1.06) | 0.752 |
| Hypertension | Income | T3 | 1.05 (1.01, 1.10) | 0.384 |
| Hypertension | Climate | Cold | 0.96 (0.91, 1.00) | Ref |
| Hypertension | Climate | Moderate cold | 1.11 (1.06, 1.17) | <0.001 |
| Hypertension | Climate | Moderate hot | 1.11 (0.99, 1.24) | 0.015 |
| Hypertension | Climate | Hot | 1.17 (1.08, 1.28) | <0.001 |
| Diabetes | Sex | Female | 0.96 (0.91, 1.00) | Ref |
| Diabetes | Sex | Male | 1.05 (0.99, 1.11) | 0.021 |
| Diabetes | Age | 60–74 yrs | 1.01 (0.97, 1.06) | Ref |
| Diabetes | Age | over 75 yrs | 0.93 (0.87, 1.01) | 0.071 |
| Diabetes | Urbanity | Urban | 1.02 (0.97, 1.06) | Ref |
| Diabetes | Urbanity | Rural | 0.93 (0.87, 1.00) | 0.038 |
| Diabetes | Education | Illiteracy and primary school | 0.98 (0.93, 1.02) | Ref |
| Diabetes | Education | High School and above | 1.02 (0.96, 1.09) | 0.243 |
| Diabetes | Marriage | Single | 0.96 (0.90, 1.03) | Ref |
| Diabetes | Marriage | Married | 1.00 (0.96, 1.05) | 0.328 |
| Diabetes | Exercise | Never | 1.01 (0.96, 1.07) | Ref |
| Diabetes | Exercise | 1–5 times/week | 0.98 (0.92, 1.05) | 0.526 |
| Diabetes | Exercise | over 6 times/week | 0.95 (0.89, 1.03) | 0.209 |
| Diabetes | Income | T1 | 0.96 (0.90, 1.03) | Ref |
| Diabetes | Income | T2 | 0.94 (0.88, 1.00) | 0.669 |
| Diabetes | Income | T3 | 1.03 (0.97, 1.10) | 0.143 |
| Diabetes | Climate | Cold | 0.97 (0.90, 1.05) | Ref |
| Diabetes | Climate | Moderate cold | 0.89 (0.82, 0.96) | 0.112 |
| Diabetes | Climate | Moderate hot | 1.31 (1.08, 1.59) | 0.005 |
| Diabetes | Climate | Hot | 1.30 (1.11, 1.54) | 0.001 |
| Cardio-cerebrovascular diseases | Sex | Female | 1.13 (1.10, 1.17) | Ref |
| Cardio-cerebrovascular diseases | Sex | Male | 1.10 (1.06, 1.14) | 0.254 |
| Cardio-cerebrovascular diseases | Age | 60–74 yrs | 1.13 (1.10, 1.16) | Ref |
| Cardio-cerebrovascular diseases | Age | over 75 yrs | 1.09 (1.04, 1.14) | 0.141 |
| Cardio-cerebrovascular diseases | Urbanity | Urban | 1.15 (1.11, 1.19) | Ref |
| Cardio-cerebrovascular diseases | Urbanity | Rural | 1.08 (1.04, 1.12) | 0.011 |
| Cardio-cerebrovascular diseases | Education | Illiteracy and primary school | 1.11 (1.08, 1.15) | Ref |
| Cardio-cerebrovascular diseases | Education | High School and above | 1.12 (1.07, 1.18) | 0.816 |
| Cardio-cerebrovascular diseases | Marriage | Single | 1.12 (1.07, 1.17) | Ref |
| Cardio-cerebrovascular diseases | Marriage | Married | 1.12 (1.09, 1.15) | 0.934 |
| Cardio-cerebrovascular diseases | Exercise | Never | 1.10 (1.06, 1.14) | Ref |
| Cardio-cerebrovascular diseases | Exercise | 1–5 times/week | 1.15 (1.10, 1.21) | 0.099 |
| Cardio-cerebrovascular diseases | Exercise | over 6 times/week | 1.10 (1.04, 1.16) | 0.991 |
| Cardio-cerebrovascular diseases | Income | T1 | 1.10 (1.06, 1.15) | Ref |
| Cardio-cerebrovascular diseases | Income | T2 | 1.10 (1.06, 1.15) | 0.929 |
| Cardio-cerebrovascular diseases | Income | T3 | 1.14 (1.09, 1.19) | 0.273 |
| Cardio-cerebrovascular diseases | Climate | Cold | 1.07 (1.02, 1.12) | Ref |
| Cardio-cerebrovascular diseases | Climate | Moderate cold | 1.22 (1.16, 1.29) | <0.001 |
| Cardio-cerebrovascular diseases | Climate | Moderate hot | 1.40 (1.23, 1.59) | <0.001 |
| Cardio-cerebrovascular diseases | Climate | Hot | 1.30 (1.17, 1.44) | 0.001 |
| Stomach diseases | Sex | Female | 1.10 (1.06, 1.14) | Ref |
| Stomach diseases | Sex | Male | 1.05 (1.01, 1.09) | 0.101 |
| Stomach diseases | Age | 60–74 yrs | 1.08 (1.05, 1.12) | Ref |
| Stomach diseases | Age | over 75 yrs | 1.07 (1.01, 1.13) | 0.681 |
| Stomach diseases | Urbanity | Urban | 1.08 (1.04, 1.13) | Ref |
| Stomach diseases | Urbanity | Rural | 1.07 (1.03, 1.11) | 0.621 |
| Stomach diseases | Education | Illiteracy and primary school | 1.10 (1.06, 1.13) | Ref |
| Stomach diseases | Education | High School and above | 1.02 (0.96, 1.08) | 0.027 |
| Stomach diseases | Marriage | Single | 1.12 (1.06, 1.18) | Ref |
| Stomach diseases | Marriage | Married | 1.06 (1.03, 1.10) | 0.117 |
| Stomach diseases | Exercise | Never | 1.07 (1.03, 1.11) | Ref |
| Stomach diseases | Exercise | 1–5 times/week | 1.07 (1.02, 1.13) | 0.908 |
| Stomach diseases | Exercise | over 6 times/week | 1.10 (1.03, 1.17) | 0.490 |
| Stomach diseases | Income | T1 | 1.14 (1.09, 1.19) | Ref |
| Stomach diseases | Income | T2 | 1.07 (1.03, 1.13) | 0.092 |
| Stomach diseases | Income | T3 | 1.01 (0.95, 1.06) | 0.001 |
| Stomach diseases | Climate | Cold | 1.00 (0.94, 1.06) | Ref |
| Stomach diseases | Climate | Moderate cold | 1.21 (1.13, 1.29) | <0.001 |
| Stomach diseases | Climate | Moderate hot | 1.36 (1.19, 1.56) | <0.001 |
| Stomach diseases | Climate | Hot | 1.24 (1.11, 1.38) | 0.001 |
| Arthritis | Sex | Female | 1.03 (1.00, 1.06) | Ref |
| Arthritis | Sex | Male | 1.02 (0.99, 1.05) | 0.569 |
| Arthritis | Age | 60–74 yrs | 1.04 (1.02, 1.07) | Ref |
| Arthritis | Age | over 75 yrs | 0.98 (0.94, 1.02) | 0.015 |
| Arthritis | Urbanity | Urban | 1.02 (0.99, 1.05) | Ref |
| Arthritis | Urbanity | Rural | 1.01 (0.98, 1.04) | 0.544 |
| Arthritis | Education | Illiteracy and primary school | 1.03 (1.00, 1.05) | Ref |
| Arthritis | Education | High School and above | 1.02 (0.97, 1.06) | 0.655 |
| Arthritis | Marriage | Single | 0.99 (0.95, 1.03) | Ref |
| Arthritis | Marriage | Married | 1.04 (1.01, 1.07) | 0.027 |
| Arthritis | Exercise | Never | 0.98 (0.96, 1.01) | Ref |
| Arthritis | Exercise | 1–5 times/week | 1.07 (1.03, 1.11) | 0.001 |
| Arthritis | Exercise | over 6 times/week | 1.03 (0.98, 1.08) | 0.118 |
| Arthritis | Income | T1 | 1.05 (1.01, 1.08) | Ref |
| Arthritis | Income | T2 | 1.02 (0.99, 1.06) | 0.388 |
| Arthritis | Income | T3 | 0.97 (0.93, 1.02) | 0.010 |
| Arthritis | Climate | Cold | 1.04 (0.99, 1.09) | Ref |
| Arthritis | Climate | Moderate cold | 1.11 (1.06, 1.16) | 0.059 |
| Arthritis | Climate | Moderate hot | 1.20 (1.08, 1.35) | 0.019 |
| Arthritis | Climate | Hot | 1.18 (1.09, 1.28) | 0.008 |
| Chronic lung diseases | Sex | Female | 1.07 (1.02, 1.12) | Ref |
| Chronic lung diseases | Sex | Male | 1.04 (0.99, 1.09) | 0.426 |
| Chronic lung diseases | Age | 60–74 yrs | 1.04 (1.00, 1.09) | Ref |
| Chronic lung diseases | Age | over 75 yrs | 1.06 (1.00, 1.13) | 0.647 |
| Chronic lung diseases | Urbanity | Urban | 1.05 (1.00, 1.11) | Ref |
| Chronic lung diseases | Urbanity | Rural | 1.05 (1.00, 1.10) | 0.925 |
| Chronic lung diseases | Education | Illiteracy and primary school | 1.05 (1.01, 1.10) | Ref |
| Chronic lung diseases | Education | High School and above | 1.04 (0.97, 1.12) | 0.817 |
| Chronic lung diseases | Marriage | Single | 1.01 (0.95, 1.08) | Ref |
| Chronic lung diseases | Marriage | Married | 1.07 (1.03, 1.12) | 0.135 |
| Chronic lung diseases | Exercise | Never | 1.05 (1.00, 1.10) | Ref |
| Chronic lung diseases | Exercise | 1–5 times/week | 1.11 (1.03, 1.19) | 0.233 |
| Chronic lung diseases | Exercise | over 6 times/week | 0.98 (0.90, 1.07) | 0.173 |
| Chronic lung diseases | Income | T1 | 1.03 (0.97, 1.09) | Ref |
| Chronic lung diseases | Income | T2 | 1.04 (0.98, 1.11) | 0.737 |
| Chronic lung diseases | Income | T3 | 1.08 (1.00, 1.17) | 0.285 |
| Chronic lung diseases | Climate | Cold | 0.91 (0.84, 0.97) | Ref |
| Chronic lung diseases | Climate | Moderate cold | 1.23 (1.13, 1.34) | <0.001 |
| Chronic lung diseases | Climate | Moderate hot | 1.09 (0.91, 1.30) | 0.061 |
| Chronic lung diseases | Climate | Hot | 1.08 (0.94, 1.23) | 0.025 |
| Asthma | Sex | Female | 1.06 (0.98, 1.13) | Ref |
| Asthma | Sex | Male | 1.13 (1.05, 1.22) | 0.196 |
| Asthma | Age | 60–74 yrs | 1.10 (1.03, 1.17) | Ref |
| Asthma | Age | over 75 yrs | 1.07 (0.98, 1.17) | 0.642 |
| Asthma | Urbanity | Urban | 1.06 (0.98, 1.14) | Ref |
| Asthma | Urbanity | Rural | 1.11 (1.03, 1.19) | 0.361 |
| Asthma | Education | Illiteracy and primary school | 1.11 (1.04, 1.17) | Ref |
| Asthma | Education | High School and above | 1.04 (0.92, 1.17) | 0.367 |
| Asthma | Marriage | Single | 1.07 (0.98, 1.17) | Ref |
| Asthma | Marriage | Married | 1.10 (1.04, 1.17) | 0.551 |
| Asthma | Exercise | Never | 1.05 (0.98, 1.12) | Ref |
| Asthma | Exercise | 1–5 times/week | 1.16 (1.05, 1.29) | 0.109 |
| Asthma | Exercise | over 6 times/week | 1.15 (1.00, 1.32) | 0.264 |
| Asthma | Income | T1 | 1.05 (0.97, 1.13) | Ref |
| Asthma | Income | T2 | 1.16 (1.06, 1.27) | 0.082 |
| Asthma | Income | T3 | 1.08 (0.96, 1.21) | 0.691 |
| Asthma | Climate | Cold | 0.91 (0.82, 1.02) | Ref |
| Asthma | Climate | Moderate cold | 1.12 (1.00, 1.26) | 0.011 |
| Asthma | Climate | Moderate hot | 1.29 (0.98, 1.70) | 0.020 |
| Asthma | Climate | Hot | 1.05 (0.86, 1.28) | 0.211 |
| Cancer | Sex | Female | 1.04 (0.90, 1.20) | Ref |
| Cancer | Sex | Male | 0.93 (0.79, 1.08) | 0.278 |
| Cancer | Age | 60–74 yrs | 1.00 (0.88, 1.12) | Ref |
| Cancer | Age | over 75 yrs | 0.92 (0.73, 1.15) | 0.532 |
| Cancer | Urbanity | Urban | 1.03 (0.90, 1.18) | Ref |
| Cancer | Urbanity | Rural | 0.91 (0.76, 1.08) | 0.255 |
| Cancer | Education | Illiteracy and primary school | 0.92 (0.81, 1.05) | Ref |
| Cancer | Education | High School and above | 1.12 (0.93, 1.34) | 0.092 |
| Cancer | Marriage | Single | 0.89 (0.72, 1.08) | Ref |
| Cancer | Marriage | Married | 1.02 (0.91, 1.16) | 0.230 |
| Cancer | Exercise | Never | 0.94 (0.81, 1.09) | Ref |
| Cancer | Exercise | 1–5 times/week | 1.10 (0.90, 1.36) | 0.227 |
| Cancer | Exercise | over 6 times/week | 0.98 (0.79, 1.23) | 0.756 |
| Cancer | Income | T1 | 0.87 (0.70, 1.07) | Ref |
| Cancer | Income | T2 | 0.99 (0.82, 1.19) | 0.352 |
| Cancer | Income | T3 | 1.11 (0.94, 1.32) | 0.066 |
| Cancer | Climate | Cold | 0.97 (0.78, 1.21) | Ref |
| Cancer | Climate | Moderate cold | 1.11 (0.88, 1.41) | 0.415 |
| Cancer | Climate | Moderate hot | 1.41 (0.89, 2.22) | 0.150 |
| Cancer | Climate | Hot | 0.89 (0.59, 1.33) | 0.713 |
| Reproductive diseases | Sex | Female | 1.09 (0.98, 1.22) | Ref |
| Reproductive diseases | Sex | Male | 1.03 (0.95, 1.12) | 0.440 |
| Reproductive diseases | Age | 60–74 yrs | 1.11 (1.02, 1.20) | Ref |
| Reproductive diseases | Age | over 75 yrs | 0.98 (0.87, 1.09) | 0.082 |
| Reproductive diseases | Urbanity | Urban | 1.08 (0.98, 1.18) | Ref |
| Reproductive diseases | Urbanity | Rural | 1.03 (0.93, 1.13) | 0.488 |
| Reproductive diseases | Education | Illiteracy and primary school | 1.07 (0.99, 1.16) | Ref |
| Reproductive diseases | Education | High School and above | 1.00 (0.89, 1.13) | 0.346 |
| Reproductive diseases | Marriage | Single | 1.07 (0.94, 1.23) | Ref |
| Reproductive diseases | Marriage | Married | 1.04 (0.97, 1.13) | 0.725 |
| Reproductive diseases | Exercise | Never | 1.11 (1.02, 1.22) | Ref |
| Reproductive diseases | Exercise | 1–5 times/week | 0.99 (0.86, 1.14) | 0.189 |
| Reproductive diseases | Exercise | over 6 times/week | 0.97 (0.84, 1.11) | 0.092 |
| Reproductive diseases | Income | T1 | 1.07 (0.96, 1.20) | Ref |
| Reproductive diseases | Income | T2 | 1.14 (1.01, 1.28) | 0.498 |
| Reproductive diseases | Income | T3 | 0.98 (0.87, 1.10) | 0.272 |
| Reproductive diseases | Climate | Cold | 0.85 (0.75, 0.97) | Ref |
| Reproductive diseases | Climate | Moderate cold | 1.49 (1.25, 1.77) | <0.001 |
| Reproductive diseases | Climate | Moderate hot | 1.49 (1.14, 1.95) | <0.001 |
| Reproductive diseases | Climate | Hot | 1.07 (0.81, 1.42) | 0.144 |

TV, temperature variability.

# Table S6. Risk (Odds ratio, OR) for all diseases associated with every 1℃ increase in TV during 2013–2014.

| Disease | Strata | Groups | Odds ratio (95% CI) | P for difference |
| --- | --- | --- | --- | --- |
| Cataract | Sex | Female | 1.05 (1.01, 1.09) | Ref |
| Cataract | Sex | Male | 1.02 (0.98, 1.07) | 0.411 |
| Cataract | Age | 60–74 yrs | 1.02 (0.99, 1.06) | Ref |
| Cataract | Age | over 75 yrs | 1.08 (1.03, 1.13) | 0.092 |
| Cataract | Urbanity | Urban | 1.06 (1.02, 1.10) | Ref |
| Cataract | Urbanity | Rural | 1.02 (0.98, 1.07) | 0.246 |
| Cataract | Education | Illiteracy and primary school | 1.06 (1.03, 1.10) | Ref |
| Cataract | Education | High School and above | 0.97 (0.92, 1.03) | 0.010 |
| Cataract | Marriage | Single | 1.06 (1.01, 1.11) | Ref |
| Cataract | Marriage | Married | 1.03 (0.99, 1.07) | 0.376 |
| Cataract | Exercise | Never | 1.05 (1.01, 1.09) | Ref |
| Cataract | Exercise | 1–5 times/week | 1.08 (1.03, 1.15) | 0.363 |
| Cataract | Exercise | over 6 times/week | 0.96 (0.90, 1.03) | 0.022 |
| Cataract | Income | T1 | 1.07 (1.02, 1.12) | Ref |
| Cataract | Income | T2 | 1.00 (0.96, 1.06) | 0.081 |
| Cataract | Income | T3 | 1.05 (0.99, 1.11) | 0.651 |
| Cataract | Climate | Cold | 0.95 (0.89, 1.01) | Ref |
| Cataract | Climate | Moderate cold | 1.04 (0.97, 1.11) | 0.038 |
| Cataract | Climate | Moderate hot | 1.19 (1.04, 1.36) | 0.002 |
| Cataract | Climate | Hot | 1.23 (1.12, 1.37) | <0.001 |
| Hypertension | Sex | Female | 1.02 (0.99, 1.05) | Ref |
| Hypertension | Sex | Male | 1.04 (1.01, 1.07) | 0.494 |
| Hypertension | Age | 60–74 yrs | 1.04 (1.01, 1.06) | Ref |
| Hypertension | Age | over 75 yrs | 1.01 (0.96, 1.05) | 0.205 |
| Hypertension | Urbanity | Urban | 1.04 (1.01, 1.07) | Ref |
| Hypertension | Urbanity | Rural | 1.01 (0.98, 1.04) | 0.162 |
| Hypertension | Education | Illiteracy and primary school | 1.03 (1.00, 1.05) | Ref |
| Hypertension | Education | High School and above | 1.03 (0.99, 1.08) | 0.764 |
| Hypertension | Marriage | Single | 1.00 (0.97, 1.04) | Ref |
| Hypertension | Marriage | Married | 1.04 (1.01, 1.07) | 0.130 |
| Hypertension | Exercise | Never | 1.03 (1.00, 1.06) | Ref |
| Hypertension | Exercise | 1–5 times/week | 1.02 (0.98, 1.06) | 0.590 |
| Hypertension | Exercise | over 6 times/week | 1.01 (0.97, 1.06) | 0.487 |
| Hypertension | Income | T1 | 1.02 (0.98, 1.06) | Ref |
| Hypertension | Income | T2 | 1.01 (0.98, 1.05) | 0.802 |
| Hypertension | Income | T3 | 1.05 (1.01, 1.09) | 0.265 |
| Hypertension | Climate | Cold | 0.96 (0.92, 1.00) | Ref |
| Hypertension | Climate | Moderate cold | 1.08 (1.03, 1.13) | <0.001 |
| Hypertension | Climate | Moderate hot | 1.07 (0.97, 1.19) | 0.051 |
| Hypertension | Climate | Hot | 1.18 (1.09, 1.27) | <0.001 |
| Diabetes | Sex | Female | 0.96 (0.92, 1.00) | Ref |
| Diabetes | Sex | Male | 1.06 (1.00, 1.12) | 0.011 |
| Diabetes | Age | 60–74 yrs | 1.02 (0.98, 1.06) | Ref |
| Diabetes | Age | over 75 yrs | 0.94 (0.87, 1.01) | 0.067 |
| Diabetes | Urbanity | Urban | 1.02 (0.98, 1.07) | Ref |
| Diabetes | Urbanity | Rural | 0.94 (0.88, 1.00) | 0.025 |
| Diabetes | Education | Illiteracy and primary school | 0.98 (0.94, 1.02) | Ref |
| Diabetes | Education | High School and above | 1.04 (0.97, 1.10) | 0.160 |
| Diabetes | Marriage | Single | 0.97 (0.90, 1.04) | Ref |
| Diabetes | Marriage | Married | 1.01 (0.97, 1.05) | 0.303 |
| Diabetes | Exercise | Never | 1.01 (0.96, 1.07) | Ref |
| Diabetes | Exercise | 1–5 times/week | 0.99 (0.93, 1.06) | 0.622 |
| Diabetes | Exercise | over 6 times/week | 0.97 (0.91, 1.04) | 0.376 |
| Diabetes | Income | T1 | 0.96 (0.90, 1.03) | Ref |
| Diabetes | Income | T2 | 0.95 (0.89, 1.01) | 0.763 |
| Diabetes | Income | T3 | 1.04 (0.98, 1.10) | 0.110 |
| Diabetes | Climate | Cold | 0.98 (0.91, 1.06) | Ref |
| Diabetes | Climate | Moderate cold | 0.88 (0.82, 0.95) | 0.053 |
| Diabetes | Climate | Moderate hot | 1.22 (1.02, 1.46) | 0.029 |
| Diabetes | Climate | Hot | 1.36 (1.17, 1.58) | <0.001 |
| Cardio-cerebrovascular diseases | Sex | Female | 1.13 (1.09, 1.16) | Ref |
| Cardio-cerebrovascular diseases | Sex | Male | 1.10 (1.06, 1.14) | 0.263 |
| Cardio-cerebrovascular diseases | Age | 60–74 yrs | 1.12 (1.09, 1.15) | Ref |
| Cardio-cerebrovascular diseases | Age | over 75 yrs | 1.08 (1.04, 1.13) | 0.188 |
| Cardio-cerebrovascular diseases | Urbanity | Urban | 1.15 (1.11, 1.18) | Ref |
| Cardio-cerebrovascular diseases | Urbanity | Rural | 1.07 (1.03, 1.11) | 0.007 |
| Cardio-cerebrovascular diseases | Education | Illiteracy and primary school | 1.11 (1.08, 1.14) | Ref |
| Cardio-cerebrovascular diseases | Education | High School and above | 1.12 (1.07, 1.17) | 0.707 |
| Cardio-cerebrovascular diseases | Marriage | Single | 1.11 (1.06, 1.16) | Ref |
| Cardio-cerebrovascular diseases | Marriage | Married | 1.11 (1.08, 1.14) | 0.962 |
| Cardio-cerebrovascular diseases | Exercise | Never | 1.09 (1.06, 1.13) | Ref |
| Cardio-cerebrovascular diseases | Exercise | 1–5 times/week | 1.15 (1.10, 1.20) | 0.086 |
| Cardio-cerebrovascular diseases | Exercise | over 6 times/week | 1.10 (1.04, 1.16) | 0.837 |
| Cardio-cerebrovascular diseases | Income | T1 | 1.09 (1.05, 1.13) | Ref |
| Cardio-cerebrovascular diseases | Income | T2 | 1.10 (1.06, 1.15) | 0.631 |
| Cardio-cerebrovascular diseases | Income | T3 | 1.14 (1.09, 1.19) | 0.124 |
| Cardio-cerebrovascular diseases | Climate | Cold | 1.06 (1.01, 1.11) | Ref |
| Cardio-cerebrovascular diseases | Climate | Moderate cold | 1.17 (1.11, 1.22) | 0.007 |
| Cardio-cerebrovascular diseases | Climate | Moderate hot | 1.32 (1.17, 1.49) | 0.001 |
| Cardio-cerebrovascular diseases | Climate | Hot | 1.26 (1.14, 1.38) | 0.002 |
| Stomach diseases | Sex | Female | 1.11 (1.07, 1.15) | Ref |
| Stomach diseases | Sex | Male | 1.06 (1.02, 1.10) | 0.083 |
| Stomach diseases | Age | 60–74 yrs | 1.09 (1.06, 1.13) | Ref |
| Stomach diseases | Age | over 75 yrs | 1.07 (1.01, 1.13) | 0.499 |
| Stomach diseases | Urbanity | Urban | 1.09 (1.05, 1.13) | Ref |
| Stomach diseases | Urbanity | Rural | 1.08 (1.04, 1.12) | 0.746 |
| Stomach diseases | Education | Illiteracy and primary school | 1.11 (1.07, 1.14) | Ref |
| Stomach diseases | Education | High School and above | 1.04 (0.98, 1.10) | 0.046 |
| Stomach diseases | Marriage | Single | 1.12 (1.07, 1.18) | Ref |
| Stomach diseases | Marriage | Married | 1.08 (1.04, 1.11) | 0.168 |
| Stomach diseases | Exercise | Never | 1.08 (1.04, 1.12) | Ref |
| Stomach diseases | Exercise | 1–5 times/week | 1.09 (1.03, 1.15) | 0.743 |
| Stomach diseases | Exercise | over 6 times/week | 1.11 (1.04, 1.18) | 0.465 |
| Stomach diseases | Income | T1 | 1.15 (1.10, 1.19) | Ref |
| Stomach diseases | Income | T2 | 1.09 (1.04, 1.14) | 0.112 |
| Stomach diseases | Income | T3 | 1.02 (0.96, 1.07) | 0.001 |
| Stomach diseases | Climate | Cold | 1.01 (0.95, 1.06) | Ref |
| Stomach diseases | Climate | Moderate cold | 1.18 (1.11, 1.26) | <0.001 |
| Stomach diseases | Climate | Moderate hot | 1.34 (1.18, 1.52) | <0.001 |
| Stomach diseases | Climate | Hot | 1.32 (1.19, 1.45) | <0.001 |
| Arthritis | Sex | Female | 1.04 (1.01, 1.07) | Ref |
| Arthritis | Sex | Male | 1.03 (1.00, 1.07) | 0.750 |
| Arthritis | Age | 60–74 yrs | 1.05 (1.03, 1.08) | Ref |
| Arthritis | Age | over 75 yrs | 0.99 (0.95, 1.04) | 0.014 |
| Arthritis | Urbanity | Urban | 1.03 (1.01, 1.06) | Ref |
| Arthritis | Urbanity | Rural | 1.02 (0.99, 1.05) | 0.483 |
| Arthritis | Education | Illiteracy and primary school | 1.04 (1.02, 1.07) | Ref |
| Arthritis | Education | High School and above | 1.03 (0.99, 1.07) | 0.611 |
| Arthritis | Marriage | Single | 1.00 (0.96, 1.04) | Ref |
| Arthritis | Marriage | Married | 1.05 (1.03, 1.08) | 0.016 |
| Arthritis | Exercise | Never | 1.00 (0.97, 1.02) | Ref |
| Arthritis | Exercise | 1–5 times/week | 1.09 (1.05, 1.13) | <0.001 |
| Arthritis | Exercise | over 6 times/week | 1.04 (1.00, 1.09) | 0.096 |
| Arthritis | Income | T1 | 1.06 (1.02, 1.09) | Ref |
| Arthritis | Income | T2 | 1.04 (1.00, 1.07) | 0.401 |
| Arthritis | Income | T3 | 0.99 (0.95, 1.03) | 0.009 |
| Arthritis | Climate | Cold | 1.05 (1.00, 1.09) | Ref |
| Arthritis | Climate | Moderate cold | 1.11 (1.06, 1.17) | 0.058 |
| Arthritis | Climate | Moderate hot | 1.24 (1.11, 1.37) | 0.004 |
| Arthritis | Climate | Hot | 1.24 (1.15, 1.33) | <0.001 |
| Chronic lung diseases | Sex | Female | 1.07 (1.02, 1.13) | Ref |
| Chronic lung diseases | Sex | Male | 1.04 (0.99, 1.09) | 0.371 |
| Chronic lung diseases | Age | 60–74 yrs | 1.05 (1.01, 1.09) | Ref |
| Chronic lung diseases | Age | over 75 yrs | 1.06 (1.00, 1.13) | 0.809 |
| Chronic lung diseases | Urbanity | Urban | 1.06 (1.00, 1.11) | Ref |
| Chronic lung diseases | Urbanity | Rural | 1.06 (1.01, 1.11) | 0.973 |
| Chronic lung diseases | Education | Illiteracy and primary school | 1.06 (1.02, 1.10) | Ref |
| Chronic lung diseases | Education | High School and above | 1.05 (0.98, 1.13) | 0.969 |
| Chronic lung diseases | Marriage | Single | 1.02 (0.96, 1.08) | Ref |
| Chronic lung diseases | Marriage | Married | 1.08 (1.03, 1.12) | 0.148 |
| Chronic lung diseases | Exercise | Never | 1.05 (1.01, 1.10) | Ref |
| Chronic lung diseases | Exercise | 1–5 times/week | 1.11 (1.04, 1.20) | 0.181 |
| Chronic lung diseases | Exercise | over 6 times/week | 0.99 (0.91, 1.07) | 0.192 |
| Chronic lung diseases | Income | T1 | 1.03 (0.98, 1.09) | Ref |
| Chronic lung diseases | Income | T2 | 1.05 (0.99, 1.12) | 0.636 |
| Chronic lung diseases | Income | T3 | 1.09 (1.01, 1.17) | 0.253 |
| Chronic lung diseases | Climate | Cold | 0.92 (0.86, 0.98) | Ref |
| Chronic lung diseases | Climate | Moderate cold | 1.20 (1.10, 1.31) | <0.001 |
| Chronic lung diseases | Climate | Moderate hot | 1.04 (0.88, 1.23) | 0.164 |
| Chronic lung diseases | Climate | Hot | 1.10 (0.97, 1.23) | 0.010 |
| Asthma | Sex | Female | 1.06 (0.99, 1.14) | Ref |
| Asthma | Sex | Male | 1.13 (1.05, 1.21) | 0.215 |
| Asthma | Age | 60–74 yrs | 1.10 (1.04, 1.17) | Ref |
| Asthma | Age | over 75 yrs | 1.07 (0.99, 1.17) | 0.584 |
| Asthma | Urbanity | Urban | 1.06 (0.98, 1.14) | Ref |
| Asthma | Urbanity | Rural | 1.11 (1.04, 1.19) | 0.347 |
| Asthma | Education | Illiteracy and primary school | 1.11 (1.05, 1.17) | Ref |
| Asthma | Education | High School and above | 1.05 (0.93, 1.17) | 0.382 |
| Asthma | Marriage | Single | 1.07 (0.98, 1.16) | Ref |
| Asthma | Marriage | Married | 1.11 (1.04, 1.18) | 0.479 |
| Asthma | Exercise | Never | 1.05 (0.99, 1.12) | Ref |
| Asthma | Exercise | 1–5 times/week | 1.17 (1.05, 1.29) | 0.092 |
| Asthma | Exercise | over 6 times/week | 1.15 (1.01, 1.32) | 0.233 |
| Asthma | Income | T1 | 1.04 (0.97, 1.12) | Ref |
| Asthma | Income | T2 | 1.16 (1.07, 1.27) | 0.059 |
| Asthma | Income | T3 | 1.09 (0.97, 1.22) | 0.553 |
| Asthma | Climate | Cold | 0.91 (0.82, 1.01) | Ref |
| Asthma | Climate | Moderate cold | 1.11 (0.99, 1.24) | 0.011 |
| Asthma | Climate | Moderate hot | 1.32 (1.02, 1.70) | 0.008 |
| Asthma | Climate | Hot | 1.05 (0.89, 1.25) | 0.158 |
| Cancer | Sex | Female | 1.05 (0.91, 1.20) | Ref |
| Cancer | Sex | Male | 0.93 (0.80, 1.08) | 0.272 |
| Cancer | Age | 60–74 yrs | 1.00 (0.89, 1.12) | Ref |
| Cancer | Age | over 75 yrs | 0.93 (0.74, 1.16) | 0.545 |
| Cancer | Urbanity | Urban | 1.03 (0.91, 1.18) | Ref |
| Cancer | Urbanity | Rural | 0.93 (0.78, 1.10) | 0.322 |
| Cancer | Education | Illiteracy and primary school | 0.93 (0.82, 1.06) | Ref |
| Cancer | Education | High School and above | 1.11 (0.94, 1.32) | 0.103 |
| Cancer | Marriage | Single | 0.88 (0.72, 1.07) | Ref |
| Cancer | Marriage | Married | 1.03 (0.92, 1.16) | 0.177 |
| Cancer | Exercise | Never | 0.95 (0.83, 1.10) | Ref |
| Cancer | Exercise | 1–5 times/week | 1.09 (0.89, 1.32) | 0.300 |
| Cancer | Exercise | over 6 times/week | 0.99 (0.80, 1.23) | 0.755 |
| Cancer | Income | T1 | 0.87 (0.71, 1.06) | Ref |
| Cancer | Income | T2 | 1.01 (0.84, 1.20) | 0.289 |
| Cancer | Income | T3 | 1.11 (0.95, 1.30) | 0.063 |
| Cancer | Climate | Cold | 0.98 (0.79, 1.22) | Ref |
| Cancer | Climate | Moderate cold | 1.04 (0.83, 1.31) | 0.709 |
| Cancer | Climate | Moderate hot | 1.33 (0.87, 2.04) | 0.216 |
| Cancer | Climate | Hot | 0.99 (0.69, 1.42) | 0.986 |
| Reproductive diseases | Sex | Female | 1.10 (0.99, 1.22) | Ref |
| Reproductive diseases | Sex | Male | 1.03 (0.95, 1.11) | 0.310 |
| Reproductive diseases | Age | 60–74 yrs | 1.10 (1.02, 1.20) | Ref |
| Reproductive diseases | Age | over 75 yrs | 0.98 (0.87, 1.09) | 0.070 |
| Reproductive diseases | Urbanity | Urban | 1.07 (0.98, 1.17) | Ref |
| Reproductive diseases | Urbanity | Rural | 1.03 (0.94, 1.14) | 0.543 |
| Reproductive diseases | Education | Illiteracy and primary school | 1.07 (0.99, 1.16) | Ref |
| Reproductive diseases | Education | High School and above | 1.00 (0.89, 1.13) | 0.332 |
| Reproductive diseases | Marriage | Single | 1.07 (0.94, 1.23) | Ref |
| Reproductive diseases | Marriage | Married | 1.04 (0.97, 1.12) | 0.723 |
| Reproductive diseases | Exercise | Never | 1.11 (1.01, 1.21) | Ref |
| Reproductive diseases | Exercise | 1–5 times/week | 1.00 (0.87, 1.14) | 0.208 |
| Reproductive diseases | Exercise | over 6 times/week | 0.97 (0.85, 1.11) | 0.098 |
| Reproductive diseases | Income | T1 | 1.07 (0.96, 1.19) | Ref |
| Reproductive diseases | Income | T2 | 1.14 (1.02, 1.28) | 0.433 |
| Reproductive diseases | Income | T3 | 0.98 (0.87, 1.09) | 0.254 |
| Reproductive diseases | Climate | Cold | 0.85 (0.75, 0.96) | Ref |
| Reproductive diseases | Climate | Moderate cold | 1.48 (1.25, 1.75) | <0.001 |
| Reproductive diseases | Climate | Moderate hot | 1.42 (1.11, 1.82) | <0.001 |
| Reproductive diseases | Climate | Hot | 1.04 (0.81, 1.33) | 0.148 |

TV, temperature variability.

# Table S7. Results of sensitivity analyses for TV 2014 using different df for mean temperature and mean relative humidity.

| Diseases | Model | Odds ratio (95% CI) | P for difference |
| --- | --- | --- | --- |
| Cataract | Primary | 1.063 (1.022, 1.105) | Ref |
| Cataract | k=4 | 1.062 (1.022, 1.104) | 0.992 |
| Cataract | k=5 | 1.062 (1.021, 1.104) | 0.980 |
| Cataract | k=6 | 1.061 (1.021, 1.103) | 0.968 |
| Cataract | k=7 | 1.061 (1.021, 1.103) | 0.952 |
| Cataract | Linear | 1.045 (1.013, 1.078) | 0.511 |
| Hypertension | Primary | 1.020 (0.992, 1.050) | Ref |
| Hypertension | k=4 | 1.021 (0.992, 1.050) | 0.978 |
| Hypertension | k=5 | 1.022 (0.993, 1.051) | 0.946 |
| Hypertension | k=6 | 1.023 (0.994, 1.052) | 0.901 |
| Hypertension | k=7 | 1.024 (0.996, 1.054) | 0.842 |
| Hypertension | Linear | 1.036 (1.012, 1.060) | 0.421 |
| Diabetes | Primary | 0.999 (0.951, 1.049) | Ref |
| Diabetes | k=4 | 0.999 (0.951, 1.049) | 0.998 |
| Diabetes | k=5 | 0.999 (0.951, 1.049) | 0.997 |
| Diabetes | k=6 | 0.999 (0.951, 1.049) | 1.000 |
| Diabetes | k=7 | 0.999 (0.952, 1.049) | 0.991 |
| Diabetes | Linear | 1.035 (0.996, 1.075) | 0.263 |
| Cardio-cerebrovascular diseases | Primary | 1.163 (1.127, 1.200) | Ref |
| Cardio-cerebrovascular diseases | k=4 | 1.163 (1.127, 1.201) | 0.979 |
| Cardio-cerebrovascular diseases | k=5 | 1.165 (1.128, 1.202) | 0.948 |
| Cardio-cerebrovascular diseases | k=6 | 1.166 (1.130, 1.203) | 0.903 |
| Cardio-cerebrovascular diseases | k=7 | 1.168 (1.132, 1.205) | 0.845 |
| Cardio-cerebrovascular diseases | Linear | 1.177 (1.147, 1.207) | 0.566 |
| Stomach diseases | Primary | 1.145 (1.104, 1.187) | Ref |
| Stomach diseases | k=4 | 1.144 (1.104, 1.187) | 0.982 |
| Stomach diseases | k=5 | 1.143 (1.103, 1.186) | 0.954 |
| Stomach diseases | k=6 | 1.142 (1.101, 1.184) | 0.917 |
| Stomach diseases | k=7 | 1.140 (1.100, 1.182) | 0.869 |
| Stomach diseases | Linear | 1.095 (1.065, 1.127) | 0.901 |
| Arthritis | Primary | 1.079 (1.050, 1.110) | Ref |
| Arthritis | k=4 | 1.079 (1.049, 1.110) | 0.984 |
| Arthritis | k=5 | 1.078 (1.049, 1.109) | 0.962 |
| Arthritis | k=6 | 1.078 (1.048, 1.108) | 0.934 |
| Arthritis | k=7 | 1.077 (1.047, 1.107) | 0.899 |
| Arthritis | Linear | 1.044 (1.021, 1.068) | 0.072 |
| Chronic lung diseases | Primary | 1.081 (1.032, 1.132) | Ref |
| Chronic lung diseases | k=4 | 1.080 (1.031, 1.131) | 0.983 |
| Chronic lung diseases | k=5 | 1.079 (1.030, 1.130) | 0.959 |
| Chronic lung diseases | k=6 | 1.078 (1.029, 1.129) | 0.931 |
| Chronic lung diseases | k=7 | 1.076 (1.028, 1.127) | 0.896 |
| Chronic lung diseases | Linear | 1.100 (1.061, 1.139) | 0.566 |
| Asthma | Primary | 1.136 (1.063, 1.215) | Ref |
| Asthma | k=4 | 1.137 (1.063, 1.215) | 0.997 |
| Asthma | k=5 | 1.137 (1.063, 1.215) | 0.995 |
| Asthma | k=6 | 1.137 (1.063, 1.215) | 0.994 |
| Asthma | k=7 | 1.137 (1.063, 1.215) | 0.996 |
| Asthma | Linear | 1.082 (1.028, 1.139) | 0.256 |
| Cancer | Primary | 0.953 (0.832, 1.093) | Ref |
| Cancer | k=4 | 0.954 (0.832, 1.093) | 0.995 |
| Cancer | k=5 | 0.955 (0.833, 1.094) | 0.986 |
| Cancer | k=6 | 0.957 (0.835, 1.096) | 0.972 |
| Cancer | k=7 | 0.959 (0.838, 1.098) | 0.952 |
| Cancer | Linear | 1.073 (0.962, 1.196) | 0.184 |
| Reproductive diseases | Primary | 1.060 (0.973, 1.155) | Ref |
| Reproductive diseases | k=4 | 1.059 (0.972, 1.154) | 0.988 |
| Reproductive diseases | k=5 | 1.058 (0.971, 1.152) | 0.970 |
| Reproductive diseases | k=6 | 1.056 (0.969, 1.150) | 0.949 |
| Reproductive diseases | k=7 | 1.054 (0.967, 1.148) | 0.924 |
| Reproductive diseases | Linear | 1.048 (0.980, 1.121) | 0.839 |

TV, temperature variability.

# Table S8. Results of sensitivity analyses for TV 2010–2014 using different df for mean temperature and mean relative humidity.

| Diseases | Model | Odds ratio (95% CI) | P for difference |
| --- | --- | --- | --- |
| Cataract | Primary | 1.034 (1.004, 1.064) | Ref |
| Cataract | k=4 | 1.034 (1.004, 1.064) | 0.995 |
| Cataract | k=5 | 1.033 (1.004, 1.064) | 0.990 |
| Cataract | k=6 | 1.033 (1.004, 1.064) | 0.983 |
| Cataract | k=7 | 1.033 (1.003, 1.063) | 0.974 |
| Cataract | Linear | 1.031 (1.003, 1.060) | 0.914 |
| Hypertension | Primary | 1.029 (1.007, 1.051) | Ref |
| Hypertension | k=4 | 1.029 (1.007, 1.052) | 0.985 |
| Hypertension | k=5 | 1.029 (1.007, 1.052) | 0.963 |
| Hypertension | k=6 | 1.030 (1.008, 1.053) | 0.935 |
| Hypertension | k=7 | 1.031 (1.009, 1.053) | 0.900 |
| Hypertension | Linear | 1.038 (1.017, 1.060) | 0.552 |
| Diabetes | Primary | 0.983 (0.947, 1.020) | Ref |
| Diabetes | k=4 | 0.983 (0.947, 1.020) | 0.994 |
| Diabetes | k=5 | 0.983 (0.947, 1.020) | 0.984 |
| Diabetes | k=6 | 0.984 (0.948, 1.021) | 0.969 |
| Diabetes | k=7 | 0.984 (0.948, 1.021) | 0.950 |
| Diabetes | Linear | 1.000 (0.967, 1.035) | 0.484 |
| Cardio-cerebrovascular diseases | Primary | 1.099 (1.073, 1.126) | Ref |
| Cardio-cerebrovascular diseases | k=4 | 1.100 (1.073, 1.127) | 0.980 |
| Cardio-cerebrovascular diseases | k=5 | 1.100 (1.074, 1.128) | 0.951 |
| Cardio-cerebrovascular diseases | k=6 | 1.101 (1.075, 1.129) | 0.913 |
| Cardio-cerebrovascular diseases | k=7 | 1.103 (1.076, 1.130) | 0.863 |
| Cardio-cerebrovascular diseases | Linear | 1.131 (1.106, 1.158) | 0.091 |
| Stomach diseases | Primary | 1.072 (1.043, 1.102) | Ref |
| Stomach diseases | k=4 | 1.072 (1.043, 1.101) | 0.985 |
| Stomach diseases | k=5 | 1.071 (1.042, 1.101) | 0.966 |
| Stomach diseases | k=6 | 1.070 (1.042, 1.100) | 0.943 |
| Stomach diseases | k=7 | 1.070 (1.041, 1.099) | 0.916 |
| Stomach diseases | Linear | 1.069 (1.042, 1.097) | 0.901 |
| Arthritis | Primary | 1.032 (1.011, 1.054) | Ref |
| Arthritis | k=4 | 1.032 (1.010, 1.054) | 0.987 |
| Arthritis | k=5 | 1.032 (1.010, 1.053) | 0.970 |
| Arthritis | k=6 | 1.031 (1.010, 1.053) | 0.950 |
| Arthritis | k=7 | 1.031 (1.009, 1.053) | 0.927 |
| Arthritis | Linear | 1.026 (1.006, 1.047) | 0.702 |
| Chronic lung diseases | Primary | 1.051 (1.016, 1.088) | Ref |
| Chronic lung diseases | k=4 | 1.051 (1.015, 1.088) | 0.992 |
| Chronic lung diseases | k=5 | 1.051 (1.015, 1.088) | 0.983 |
| Chronic lung diseases | k=6 | 1.051 (1.015, 1.087) | 0.974 |
| Chronic lung diseases | k=7 | 1.050 (1.015, 1.087) | 0.965 |
| Chronic lung diseases | Linear | 1.073 (1.039, 1.108) | 0.397 |
| Asthma | Primary | 1.100 (1.046, 1.157) | Ref |
| Asthma | k=4 | 1.100 (1.046, 1.157) | 0.996 |
| Asthma | k=5 | 1.099 (1.045, 1.156) | 0.990 |
| Asthma | k=6 | 1.099 (1.045, 1.156) | 0.981 |
| Asthma | k=7 | 1.098 (1.045, 1.155) | 0.970 |
| Asthma | Linear | 1.089 (1.040, 1.141) | 0.778 |
| Cancer | Primary | 0.981 (0.884, 1.089) | Ref |
| Cancer | k=4 | 0.982 (0.884, 1.089) | 0.992 |
| Cancer | k=5 | 0.983 (0.886, 1.090) | 0.980 |
| Cancer | k=6 | 0.984 (0.887, 1.092) | 0.965 |
| Cancer | k=7 | 0.986 (0.889, 1.093) | 0.945 |
| Cancer | Linear | 1.034 (0.937, 1.140) | 0.470 |
| Reproductive diseases | Primary | 1.038 (0.972, 1.108) | Ref |
| Reproductive diseases | k=4 | 1.037 (0.972, 1.107) | 0.991 |
| Reproductive diseases | k=5 | 1.037 (0.972, 1.106) | 0.981 |
| Reproductive diseases | k=6 | 1.036 (0.971, 1.105) | 0.969 |
| Reproductive diseases | k=7 | 1.035 (0.971, 1.104) | 0.957 |
| Reproductive diseases | Linear | 1.044 (0.982, 1.109) | 0.899 |

TV, temperature variability.

# Table S9. Results of sensitivity analyses for TV 2011–2014 using different df for mean temperature and mean relative humidity.

| Diseases | Model | Odds ratio (95% CI) | P for difference |
| --- | --- | --- | --- |
| Cataract | Primary | 1.036 (1.007, 1.066) | Ref |
| Cataract | k=4 | 1.036 (1.006, 1.066) | 0.995 |
| Cataract | k=5 | 1.036 (1.006, 1.066) | 0.989 |
| Cataract | k=6 | 1.035 (1.006, 1.065) | 0.983 |
| Cataract | k=7 | 1.035 (1.006, 1.065) | 0.974 |
| Cataract | Linear | 1.034 (1.006, 1.063) | 0.925 |
| Hypertension | Primary | 1.031 (1.009, 1.053) | Ref |
| Hypertension | k=4 | 1.032 (1.010, 1.054) | 0.986 |
| Hypertension | k=5 | 1.032 (1.010, 1.054) | 0.966 |
| Hypertension | k=6 | 1.032 (1.011, 1.055) | 0.939 |
| Hypertension | k=7 | 1.033 (1.011, 1.055) | 0.907 |
| Hypertension | Linear | 1.040 (1.019, 1.061) | 0.591 |
| Diabetes | Primary | 0.986 (0.951, 1.022) | Ref |
| Diabetes | k=4 | 0.986 (0.951, 1.023) | 0.995 |
| Diabetes | k=5 | 0.986 (0.951, 1.023) | 0.986 |
| Diabetes | k=6 | 0.987 (0.952, 1.023) | 0.973 |
| Diabetes | k=7 | 0.987 (0.952, 1.024) | 0.956 |
| Diabetes | Linear | 1.003 (0.969, 1.037) | 0.508 |
| Cardio-cerebrovascular diseases | Primary | 1.099 (1.073, 1.125) | Ref |
| Cardio-cerebrovascular diseases | k=4 | 1.099 (1.073, 1.126) | 0.979 |
| Cardio-cerebrovascular diseases | k=5 | 1.100 (1.074, 1.126) | 0.951 |
| Cardio-cerebrovascular diseases | k=6 | 1.101 (1.075, 1.127) | 0.912 |
| Cardio-cerebrovascular diseases | k=7 | 1.102 (1.076, 1.129) | 0.861 |
| Cardio-cerebrovascular diseases | Linear | 1.129 (1.104, 1.154) | 0.109 |
| Stomach diseases | Primary | 1.067 (1.038, 1.096) | Ref |
| Stomach diseases | k=4 | 1.066 (1.038, 1.095) | 0.987 |
| Stomach diseases | k=5 | 1.066 (1.038, 1.095) | 0.971 |
| Stomach diseases | k=6 | 1.065 (1.037, 1.094) | 0.950 |
| Stomach diseases | k=7 | 1.065 (1.037, 1.094) | 0.927 |
| Stomach diseases | Linear | 1.064 (1.038, 1.092) | 0.901 |
| Arthritis | Primary | 1.029 (1.008, 1.051) | Ref |
| Arthritis | k=4 | 1.029 (1.008, 1.051) | 0.988 |
| Arthritis | k=5 | 1.029 (1.008, 1.050) | 0.973 |
| Arthritis | k=6 | 1.029 (1.008, 1.050) | 0.956 |
| Arthritis | k=7 | 1.028 (1.007, 1.050) | 0.935 |
| Arthritis | Linear | 1.024 (1.004, 1.044) | 0.712 |
| Chronic lung diseases | Primary | 1.050 (1.015, 1.087) | Ref |
| Chronic lung diseases | k=4 | 1.050 (1.015, 1.087) | 0.993 |
| Chronic lung diseases | k=5 | 1.050 (1.015, 1.086) | 0.985 |
| Chronic lung diseases | k=6 | 1.050 (1.014, 1.086) | 0.977 |
| Chronic lung diseases | k=7 | 1.049 (1.014, 1.085) | 0.970 |
| Chronic lung diseases | Linear | 1.071 (1.037, 1.106) | 0.414 |
| Asthma | Primary | 1.098 (1.045, 1.154) | Ref |
| Asthma | k=4 | 1.098 (1.045, 1.154) | 0.996 |
| Asthma | k=5 | 1.098 (1.044, 1.154) | 0.990 |
| Asthma | k=6 | 1.097 (1.044, 1.153) | 0.982 |
| Asthma | k=7 | 1.097 (1.044, 1.152) | 0.971 |
| Asthma | Linear | 1.087 (1.038, 1.139) | 0.776 |
| Cancer | Primary | 0.980 (0.885, 1.086) | Ref |
| Cancer | k=4 | 0.981 (0.886, 1.087) | 0.992 |
| Cancer | k=5 | 0.982 (0.887, 1.087) | 0.981 |
| Cancer | k=6 | 0.983 (0.888, 1.089) | 0.965 |
| Cancer | k=7 | 0.985 (0.890, 1.090) | 0.946 |
| Cancer | Linear | 1.031 (0.936, 1.136) | 0.483 |
| Reproductive diseases | Primary | 1.034 (0.970, 1.102) | Ref |
| Reproductive diseases | k=4 | 1.034 (0.970, 1.102) | 0.992 |
| Reproductive diseases | k=5 | 1.033 (0.969, 1.101) | 0.983 |
| Reproductive diseases | k=6 | 1.032 (0.969, 1.100) | 0.973 |
| Reproductive diseases | k=7 | 1.032 (0.968, 1.099) | 0.963 |
| Reproductive diseases | Linear | 1.039 (0.978, 1.103) | 0.919 |

TV, temperature variability.

# Table S10. Results of sensitivity analyses for TV 2012–2014 using different df for mean temperature and mean relative humidity.

| Diseases | Model | Odds ratio (95% CI) | P for difference |
| --- | --- | --- | --- |
| Cataract | Primary | 1.044 (1.014, 1.075) | Ref |
| Cataract | k=4 | 1.044 (1.014, 1.075) | 0.995 |
| Cataract | k=5 | 1.044 (1.014, 1.075) | 0.988 |
| Cataract | k=6 | 1.044 (1.014, 1.075) | 0.981 |
| Cataract | k=7 | 1.043 (1.013, 1.074) | 0.971 |
| Cataract | Linear | 1.041 (1.013, 1.071) | 0.898 |
| Hypertension | Primary | 1.035 (1.013, 1.058) | Ref |
| Hypertension | k=4 | 1.035 (1.013, 1.058) | 0.987 |
| Hypertension | k=5 | 1.036 (1.014, 1.059) | 0.967 |
| Hypertension | k=6 | 1.036 (1.014, 1.059) | 0.942 |
| Hypertension | k=7 | 1.037 (1.015, 1.060) | 0.910 |
| Hypertension | Linear | 1.044 (1.022, 1.065) | 0.594 |
| Diabetes | Primary | 0.991 (0.955, 1.029) | Ref |
| Diabetes | k=4 | 0.991 (0.955, 1.029) | 0.996 |
| Diabetes | k=5 | 0.991 (0.956, 1.029) | 0.988 |
| Diabetes | k=6 | 0.992 (0.956, 1.029) | 0.977 |
| Diabetes | k=7 | 0.992 (0.957, 1.029) | 0.961 |
| Diabetes | Linear | 1.008 (0.974, 1.043) | 0.510 |
| Cardio-cerebrovascular diseases | Primary | 1.117 (1.090, 1.145) | Ref |
| Cardio-cerebrovascular diseases | k=4 | 1.117 (1.091, 1.145) | 0.982 |
| Cardio-cerebrovascular diseases | k=5 | 1.118 (1.091, 1.146) | 0.957 |
| Cardio-cerebrovascular diseases | k=6 | 1.119 (1.092, 1.146) | 0.923 |
| Cardio-cerebrovascular diseases | k=7 | 1.120 (1.093, 1.147) | 0.878 |
| Cardio-cerebrovascular diseases | Linear | 1.146 (1.120, 1.172) | 0.135 |
| Stomach diseases | Primary | 1.078 (1.049, 1.108) | Ref |
| Stomach diseases | k=4 | 1.077 (1.048, 1.107) | 0.986 |
| Stomach diseases | k=5 | 1.077 (1.048, 1.107) | 0.967 |
| Stomach diseases | k=6 | 1.076 (1.047, 1.106) | 0.945 |
| Stomach diseases | k=7 | 1.076 (1.047, 1.105) | 0.918 |
| Stomach diseases | Linear | 1.075 (1.047, 1.103) | 0.901 |
| Arthritis | Primary | 1.025 (1.003, 1.046) | Ref |
| Arthritis | k=4 | 1.024 (1.003, 1.046) | 0.989 |
| Arthritis | k=5 | 1.024 (1.003, 1.046) | 0.976 |
| Arthritis | k=6 | 1.024 (1.003, 1.046) | 0.959 |
| Arthritis | k=7 | 1.023 (1.002, 1.045) | 0.941 |
| Arthritis | Linear | 1.020 (1.000, 1.041) | 0.769 |
| Chronic lung diseases | Primary | 1.052 (1.016, 1.090) | Ref |
| Chronic lung diseases | k=4 | 1.052 (1.016, 1.089) | 0.992 |
| Chronic lung diseases | k=5 | 1.052 (1.015, 1.089) | 0.984 |
| Chronic lung diseases | k=6 | 1.051 (1.015, 1.089) | 0.975 |
| Chronic lung diseases | k=7 | 1.051 (1.015, 1.088) | 0.966 |
| Chronic lung diseases | Linear | 1.074 (1.040, 1.110) | 0.392 |
| Asthma | Primary | 1.092 (1.038, 1.149) | Ref |
| Asthma | k=4 | 1.092 (1.037, 1.149) | 0.996 |
| Asthma | k=5 | 1.091 (1.037, 1.148) | 0.991 |
| Asthma | k=6 | 1.091 (1.037, 1.148) | 0.984 |
| Asthma | k=7 | 1.091 (1.037, 1.147) | 0.975 |
| Asthma | Linear | 1.083 (1.033, 1.135) | 0.814 |
| Cancer | Primary | 0.981 (0.883, 1.088) | Ref |
| Cancer | k=4 | 0.981 (0.884, 1.089) | 0.992 |
| Cancer | k=5 | 0.982 (0.885, 1.090) | 0.981 |
| Cancer | k=6 | 0.984 (0.887, 1.091) | 0.966 |
| Cancer | k=7 | 0.985 (0.888, 1.093) | 0.947 |
| Cancer | Linear | 1.032 (0.936, 1.139) | 0.481 |
| Reproductive diseases | Primary | 1.051 (0.984, 1.122) | Ref |
| Reproductive diseases | k=4 | 1.050 (0.984, 1.121) | 0.991 |
| Reproductive diseases | k=5 | 1.049 (0.983, 1.120) | 0.980 |
| Reproductive diseases | k=6 | 1.049 (0.983, 1.119) | 0.967 |
| Reproductive diseases | k=7 | 1.048 (0.982, 1.118) | 0.954 |
| Reproductive diseases | Linear | 1.053 (0.991, 1.120) | 0.955 |

TV, temperature variability.

# Table S11. Results of sensitivity analyses for TV 2013–2014 using different df for mean temperature and mean relative humidity.

| Diseases | Model | Odds ratio (95% CI) | P for difference |
| --- | --- | --- | --- |
| Cataract | Primary | 1.039 (1.010, 1.069) | Ref |
| Cataract | k=4 | 1.039 (1.010, 1.069) | 0.996 |
| Cataract | k=5 | 1.039 (1.010, 1.069) | 0.990 |
| Cataract | k=6 | 1.039 (1.010, 1.069) | 0.984 |
| Cataract | k=7 | 1.039 (1.010, 1.069) | 0.977 |
| Cataract | Linear | 1.037 (1.009, 1.066) | 0.907 |
| Hypertension | Primary | 1.029 (1.007, 1.050) | Ref |
| Hypertension | k=4 | 1.029 (1.007, 1.051) | 0.988 |
| Hypertension | k=5 | 1.029 (1.008, 1.051) | 0.971 |
| Hypertension | k=6 | 1.030 (1.008, 1.051) | 0.947 |
| Hypertension | k=7 | 1.030 (1.009, 1.052) | 0.918 |
| Hypertension | Linear | 1.037 (1.016, 1.058) | 0.595 |
| Diabetes | Primary | 0.997 (0.962, 1.033) | Ref |
| Diabetes | k=4 | 0.997 (0.962, 1.033) | 0.998 |
| Diabetes | k=5 | 0.997 (0.962, 1.033) | 0.993 |
| Diabetes | k=6 | 0.997 (0.962, 1.033) | 0.986 |
| Diabetes | k=7 | 0.998 (0.963, 1.034) | 0.975 |
| Diabetes | Linear | 1.012 (0.978, 1.046) | 0.551 |
| Cardio-cerebrovascular diseases | Primary | 1.110 (1.085, 1.137) | Ref |
| Cardio-cerebrovascular diseases | k=4 | 1.111 (1.085, 1.137) | 0.985 |
| Cardio-cerebrovascular diseases | k=5 | 1.111 (1.086, 1.138) | 0.963 |
| Cardio-cerebrovascular diseases | k=6 | 1.112 (1.086, 1.138) | 0.932 |
| Cardio-cerebrovascular diseases | k=7 | 1.113 (1.087, 1.139) | 0.892 |
| Cardio-cerebrovascular diseases | Linear | 1.137 (1.112, 1.163) | 0.152 |
| Stomach diseases | Primary | 1.088 (1.059, 1.117) | Ref |
| Stomach diseases | k=4 | 1.088 (1.059, 1.117) | 0.988 |
| Stomach diseases | k=5 | 1.087 (1.058, 1.117) | 0.971 |
| Stomach diseases | k=6 | 1.087 (1.058, 1.116) | 0.951 |
| Stomach diseases | k=7 | 1.086 (1.057, 1.115) | 0.926 |
| Stomach diseases | Linear | 1.084 (1.057, 1.112) | 0.901 |
| Arthritis | Primary | 1.037 (1.016, 1.059) | Ref |
| Arthritis | k=4 | 1.037 (1.016, 1.058) | 0.990 |
| Arthritis | k=5 | 1.037 (1.016, 1.058) | 0.977 |
| Arthritis | k=6 | 1.036 (1.015, 1.058) | 0.961 |
| Arthritis | k=7 | 1.036 (1.015, 1.057) | 0.942 |
| Arthritis | Linear | 1.032 (1.012, 1.052) | 0.730 |
| Chronic lung diseases | Primary | 1.056 (1.021, 1.093) | Ref |
| Chronic lung diseases | k=4 | 1.056 (1.021, 1.093) | 0.993 |
| Chronic lung diseases | k=5 | 1.056 (1.020, 1.092) | 0.984 |
| Chronic lung diseases | k=6 | 1.055 (1.020, 1.092) | 0.975 |
| Chronic lung diseases | k=7 | 1.055 (1.020, 1.091) | 0.965 |
| Chronic lung diseases | Linear | 1.076 (1.042, 1.111) | 0.441 |
| Asthma | Primary | 1.093 (1.040, 1.149) | Ref |
| Asthma | k=4 | 1.093 (1.040, 1.148) | 0.998 |
| Asthma | k=5 | 1.093 (1.040, 1.148) | 0.994 |
| Asthma | k=6 | 1.093 (1.040, 1.148) | 0.989 |
| Asthma | k=7 | 1.092 (1.040, 1.147) | 0.983 |
| Asthma | Linear | 1.085 (1.036, 1.137) | 0.835 |
| Cancer | Primary | 0.987 (0.892, 1.092) | Ref |
| Cancer | k=4 | 0.987 (0.893, 1.092) | 0.994 |
| Cancer | k=5 | 0.988 (0.894, 1.093) | 0.985 |
| Cancer | k=6 | 0.989 (0.895, 1.094) | 0.973 |
| Cancer | k=7 | 0.991 (0.896, 1.095) | 0.957 |
| Cancer | Linear | 1.033 (0.938, 1.137) | 0.522 |
| Reproductive diseases | Primary | 1.049 (0.984, 1.118) | Ref |
| Reproductive diseases | k=4 | 1.049 (0.984, 1.117) | 0.993 |
| Reproductive diseases | k=5 | 1.048 (0.984, 1.117) | 0.984 |
| Reproductive diseases | k=6 | 1.047 (0.983, 1.116) | 0.973 |
| Reproductive diseases | k=7 | 1.047 (0.983, 1.115) | 0.961 |
| Reproductive diseases | Linear | 1.050 (0.989, 1.115) | 0.976 |

TV, temperature variability.
